# Structural basis for simvastatin-induced skeletal muscle weakness associated with type 1 ryanodine receptor T4709M mutation

**DOI:** 10.1172/JCI194490

**Published:** 2025-12-15

**Authors:** Gunnar Weninger, Haikel Dridi, Steven Reiken, Qi Yuan, Nan Zhao, Linda Groom, Jennifer Leigh, Yang Liu, Carl Tchagou, Jiayi Kang, Alexander Chang, Estefania Luna-Figueroa, Marco C. Miotto, Anetta Wronska, Robert T. Dirksen, Andrew R. Marks

**Affiliations:** 1 Department of Physiology and Cellular Biophysics, Clyde and Helen Wu Center for Molecular Cardiology, Vagelos College of Physicians and Surgeons, Columbia University Medical Center, New York, New York, USA.; 2 Department of Pharmacology and Physiology, University of Rochester Medical Center, Rochester, New York, USA.

**Keywords:** Cell biology, Metabolism, Muscle biology, Calcium, Drug therapy, Ion channels

## Abstract

Statins lower cholesterol, reducing the risk of heart disease, and are among the most frequently prescribed drugs. Approximately 10% of individuals develop statin-associated muscle symptoms (SAMS; myalgias, rhabdomyolysis, and muscle weakness), often rendering them statin intolerant. The mechanism underlying SAMS remains poorly understood. Patients with mutations in the skeletal muscle ryanodine receptor 1 (RyR1)/calcium release channel can be particularly intolerant of statins. High-resolution structures revealed simvastatin binding sites in the pore region of RyR1. Simvastatin stabilized the open conformation of the pore and activated the RyR1 channel. In a mouse expressing a mutant RyR1-T4709M found in a patient with profound statin intolerance, simvastatin caused muscle weakness associated with leaky RyR1 channels. Cotreatment with a Rycal drug that stabilizes the channel closed state prevented simvastatin-induced muscle weakness. Thus, statin binding to RyR1 can cause SAMS, and patients with RyR1 mutations may represent a high-risk group for statin intolerance.

## Abstract 2

Statin side effects on skeletal muscle are associated with <span>altered</span> calcium signaling. We provide structural and functional insights into how simvastatin impacts calcium release channels.

## Graphical abstract

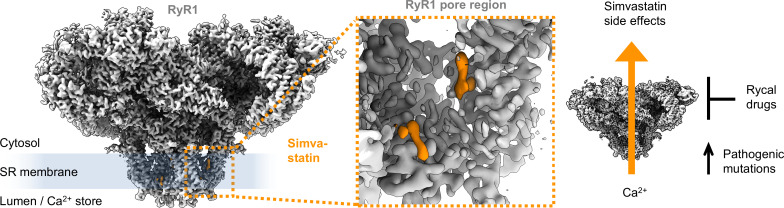

## Introduction

Statins are among the most frequently prescribed drugs as first-line treatments to lower cholesterol levels. Statin therapies reduce the risk of cardiovascular diseases linked to elevated LDL cholesterol levels (1–3). Statin drugs inhibit HMG-CoA reductase, the rate-limiting enzyme in the mevalonate pathway required for de novo cholesterol biosynthesis in the liver (1, 2). HMG-CoA reductase inhibition is mediated by the open acid form of the statin lactone group that forms an HMG-like moiety (Supplemental Figure 1; supplemental material available online with this article; https://doi.org/10.1172/JCI194490DS1), blocking the substrate access to the active site of the enzyme, while statins with closed-ring lactone groups are inactive prodrugs (4). Statin prodrugs are reversibly hydrolyzed into the active acid form by carboxylesterases in the liver (5). Moreover, statin-mediated reduction in cholesterol biosynthesis can cause low intrahepatic cholesterol levels that upregulate LDL receptor activity, and thus uptake of LDL cholesterol from the blood, further lowering plasma cholesterol levels (1, 2).

Statin side effects are presented in ~10% of treated individuals (6) and commonly include skeletal muscle complaints varying from mild cases of myalgia along with muscle weakness, stiffness, tenderness, and cramps to rare but life-threatening cases of rhabdomyolysis culminating in autoimmune-mediated necrotizing myositis (7–9). These statin-associated muscle symptoms (SAMS) typically affect the proximal musculature in the lower limbs (10).

Blockade of the mevalonate pathway by statins may directly affect several branching metabolic pathways in addition to cholesterol synthesis, resulting in coenzyme Q_10_ deficiency and loss of protein prenylation, both of which may contribute to SAMS (11–13). However, statin lactone prodrugs, inactive as HMG-CoA reductase inhibitors, have been associated with adverse drug reactions, suggesting the existence of off-target mechanisms that account for SAMS (14–16). Moreover, SAMS have been linked to statin-induced intracellular Ca^2+^ release, impacting Ca^2+^ signaling pathways in skeletal muscle (17, 18). Nevertheless, the molecular mechanism(s) that underlies SAMS remains poorly understood (13, 16).

Profound statin intolerance was previously found in a patient carrying a heterozygous T4709M (TM) mutation in the type 1 ryanodine receptor (RyR1) (19). RyR1 is the primary Ca^2+^ release channel of the sarcoplasmic reticulum (SR) in skeletal muscle, where SR Ca^2+^ release plays a crucial role in triggering muscle contractions (20, 21). Pathogenic mutations that render RyR1 channels dysfunctional are linked to malignant hyperthermia (MH) (22), exercise-induced rhabdomyolysis (23), and a wide range of RyR1-related myopathies (RyR1-RMs), including central core disease (CCD) (24). Interestingly, TM was characterized as a pathogenic mutation that does not cause Ca^2+^ leak under resting conditions but rather sensitizes RyR1 to activating conditions (19). Although TM represents a recessive mutation with low prevalence, silencing of the second RyR1 gene allele by compound heterozygosity can lead to MH or CCD-like phenotypes observed in animal models (25) and patients (19). The TM mutation may enhance the sensitivity of RyR1 to activation by various triggers (e.g., voltage sensor, caffeine, halothane, and 4-chloro-m-cresol) (19, 25), potentially leading to intolerances to clinically relevant drugs.

Importantly, recent reports suggest that RyR1-RMs can underlie sensitization to statin drugs, including simvastatin (17, 26, 27). Such a pathogenic condition is presented by the Y522S (YS) mutation of RyR1, which is associated with RyR1-RM disorders in patients (27, 28). The YS mutation knockin mouse model of RyR1-RM, which destabilizes the RyR1 resting closed state, was reported to be more susceptible to simvastatin-induced adverse effects on skeletal muscles exhibiting increased SR Ca^2+^ leak, abnormal muscle contractures, and stronger MH reactions upon simvastatin treatments compared with WT (27), while effects of statins on other pathogenic RyR1 mutants, including TM, have yet to be evaluated. Indeed, RyR1 channels have been identified as potential statin off-target effectors in vitro in single-channel recordings and in [^3^H]ryanodine binding assays (14, 29).

Using cryogenic electron microscopy (cryo-EM), previous structural studies revealed the homotetrameric architecture of the 2.3 MDa RyR1 channels exhibiting a mushroom-like shape that consists of a huge cytosolic shell (head) and a C-terminal pore region (stalk) spanning the SR membrane (30–32). While the cytosolic shell is mainly formed by a large α-solenoid network interspersed with globular domains, the transmembrane domain (TMD) possesses a 6-transmembrane-helix architecture (helices S1–S6) typical for cation channels, including a pseudo-voltage sensor domain (pVSD) (S1–S4) and the inner pore (S5–S6) (33). In the present study, we took advantage of recently improved purification protocols and cryo-EM workflows (34, 35) to resolve simvastatin binding sites in the pore region of RyR1 at near-atomic resolutions, revealing structural insights into simvastatin-induced muscle toxicities based on binding to RyR1.

## Results

### Structure of RyR1 with simvastatin.

To solve the structure of simvastatin binding sites on RyR1, we chromatographically purified endogenous RyR1 channels from mouse skeletal muscle tissues. RyR1 complexes with simvastatin were formed by adding ~10 mM simvastatin lactone to purified RyR1 (protomers: ~15 μM final). The solvent DMSO (~1% final) used to dissolve the hydrophobic simvastatin lactone did not substantially affect RyR1 activity, as confirmed in single-channel recordings (Supplemental Figure 2). RyR1 inhibition was previously reported in single-channel recordings at DMSO concentrations exceeding 2% (36). To facilitate RyR1 pore opening, the RyR1-activating ligands ATP (10 mM), caffeine (5 mM), and Ca^2+^ (30 μM free) were added, as described previously for rabbit RyR1 (33). After vitrification, RyR1 particles in the samples were assessed by cryo-EM single-particle analysis (Supplemental Figure 3 and Supplemental Table 1). The activating conditions enabled separation of closed and open conformations of RyR1 via 3D classification using CryoSPARC software (Supplemental Figure 3, A and B). Following nonuniform and local refinements (cryoSPARC), the structure of RyR1 with simvastatin was reconstructed at near-atomic resolutions reaching ~2.6 Å overall and ~2.4 Å locally at the pore in the closed conformation (*N* = 65,309 particles), while the open conformational state (*N* = 25,791 particles) was solved at ~3.1 Å global resolution and ~2.9 Å local resolution at the pore (Supplemental Figure 4). Improved resolutions were obtained for the RyR1 structure without simvastatin: ~2.4 Å global and ~2.2 Å local resolution at the pore for the closed conformation (*N* = 224,439 particles) as well as ~3.2 Å global and ~2.9 Å local resolution at the pore for the open conformation (*N* = 26,588 particles). Interestingly, the ratio of open to closed RyR1 particles increased from ~0.12 to ~0.40 in the presence of simvastatin.

In absence of simvastatin and activating ligands, an apo-closed structure of RyR1 was reconstructed from particles under EGTA conditions (*N* = 129,073 particles) solved globally at ~2.9 Å and locally at ~2.7 Å resolution in the pore region (Supplemental Figure 3C) exhibiting the unstable auxiliary transmembrane helix TMx (residues 4,318–4,340 in mouse), which is absent in the other RyR1 structures described here. Indeed, the TMx helix is frequently not observed in RyR1 structures presumably due to denaturation of TMx during cell lysis or solubilization using the zwitterionic detergent 3-((3-cholamidopropyl)dimethylammonio)-1-propanesulfonate (CHAPS) (33). Denaturation of TMx may expose potentially artificial hydrophobic interaction sites inside the TMD (Supplemental Figure 5).

**Figure 1:**
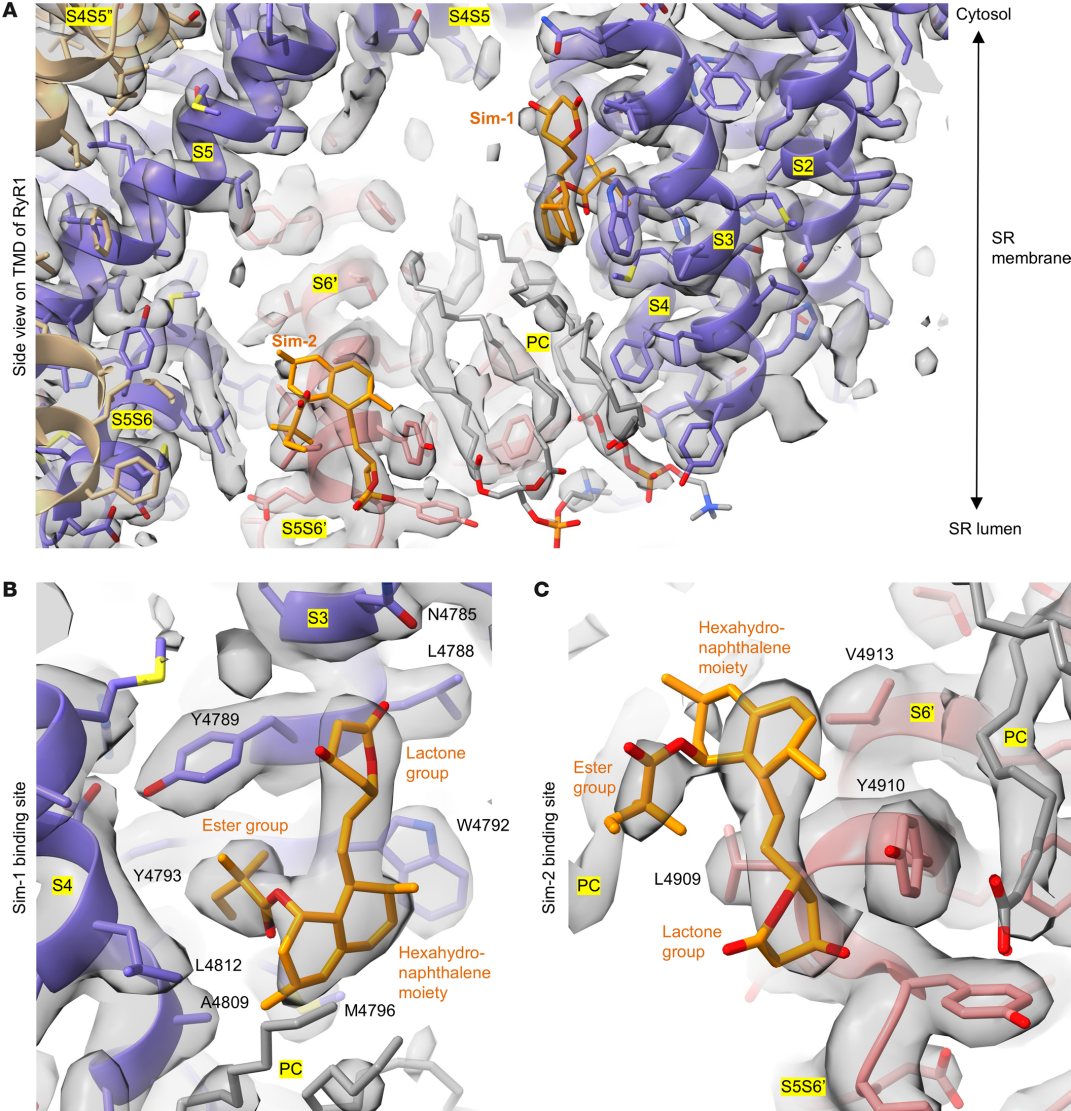
Simvastatin binding sites in the TMD of RyR1. (**A**) Side view of TMD of RyR1 (PDB: 9NMQ) including helices S1–S6 (neighboring protomers colored in purple and red), phosphatidylcholine (PC) (gray), and simvastatin (orange). (**B**) Simvastatin binding site Sim-1 located between transmembrane helices S3 and S4 of RyR1. Residues Y4789, Y4793, and M4796 of S3 and L4812 of S4 form a hydrophobic pocket into which the 2,2-dimethylbutyrate ester group of simvastatin protrudes. The lactone group of simvastatin interacts with residues Y4789, N4785, and L4788 of S3 near the cytosolic TMD surface. The hexahydronaphthalene moiety of simvastatin makes contacts with W4792 of S3, A4809 and L4812 of S4, and putatively with acyl chains of PC lipids. (**C**) The second simvastatin (Sim-2) binds to the S6 helix near the S5S6 loop. Its hexahydronaphthalene moiety interacts with V4913, the 2,2-dimethylbutyrate ester group with L4909, and the lactone group with Y4910 near the luminal TMD surface.

We compared the cryo-EM maps of RyR1 with and without simvastatin and identified 2 distinct densities for simvastatin per protomer situated in the pore region of RyR1: simvastatin binding sites Sim-1 and Sim-2 (Figure 1A). The well-defined densities of Sim-1 and Sim-2 enabled accurate determination of both simvastatin binding poses on RyR1 and derivation of atomic models for the RyR1-simvastatin interactions in closed and open RyR1 states (Protein Data Bank [PDB]: 9NMQ and 9NMP, respectively). As shown by structural comparisons, both occupied simvastatin binding sites remained essentially unaltered in closed versus open pore conformations (Supplemental Figure 6).

**Figure 2:**
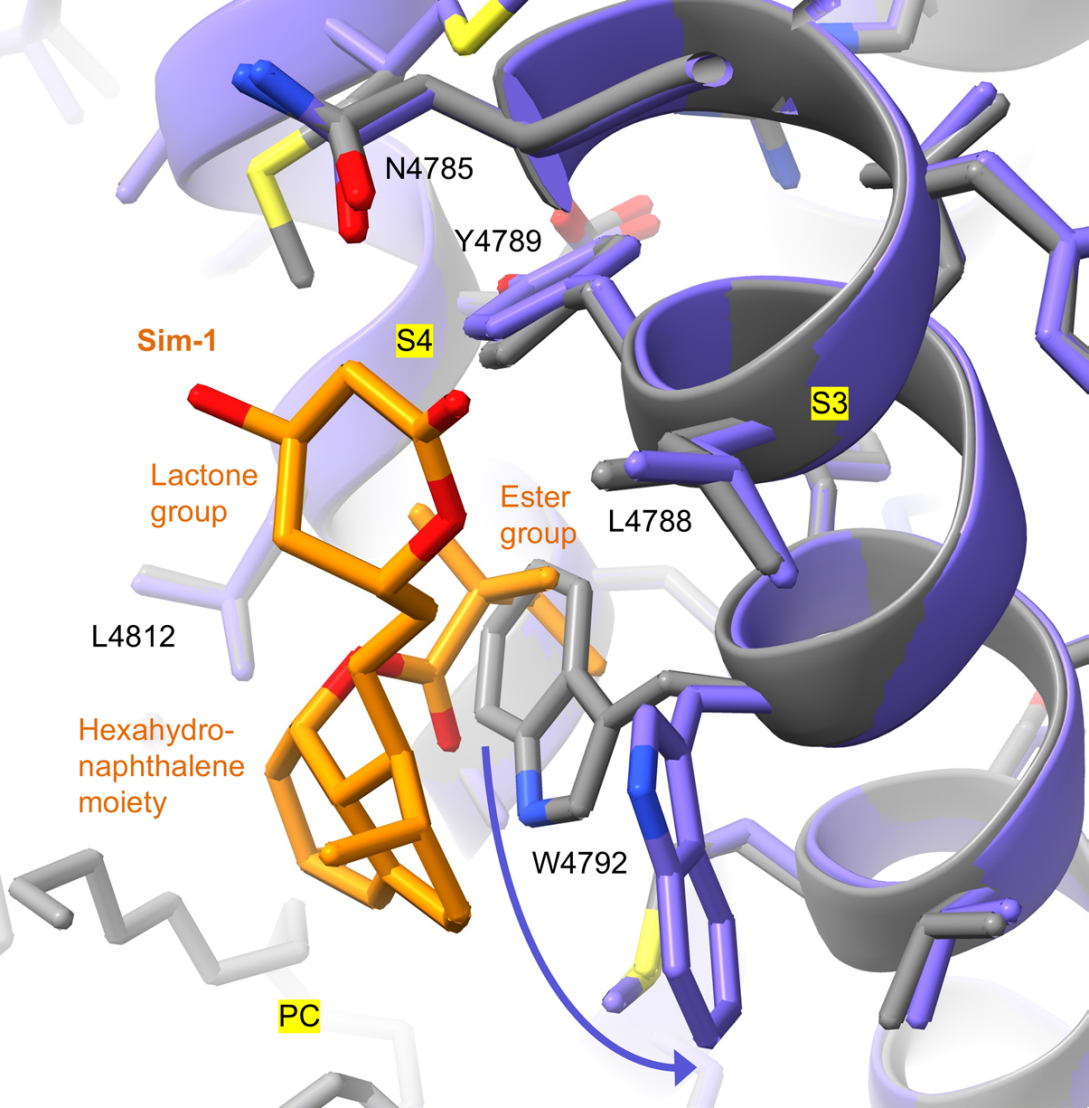
Side chain conformational change of W4792 exposes a hydrophobic pocket between transmembrane helices S3 and S4, enabling simvastatin binding at Sim-1. Structural comparison of RyR1 without (gray; PDB: 9NMO) versus with simvastatin (purple; PDB: 9NMQ) reveals that the 2,2-dimethylbutyrate ester group of simvastatin displaces the side chain of W4792. As a result, a hydrophobic pocket opens between S3 and S4, mediating tight interactions with simvastatin. PC, phosphatidylcholine.

In the RyR1-simvastatin structures, Sim-1 is located between transmembrane helices S3 and S4 of pVSD in the TMD (Figure 1 and Supplemental Figure 7). To access Sim-1, the 2,2-dimethylbutyrate ester group of simvastatin displaces the side chain of W4792 (Figure 2). As a result, a hydrophobic pocket opens between residues Y4789, Y4793, and M4796 of S3 and L4812 of S4, where the 2,2-dimethylbutyrate ester group of simvastatin protrudes (Figure 1B). Near the cytosolic TMD surface, the lactone group of simvastatin makes contacts with residues Y4789, N4785, and L4788 of S3 (Figure 1B). Inside the hydrophobic core region of TMD, the hexahydronaphthalene moiety of simvastatin interacts with W4792 of S3, A4809 of S4, and L4812 of S4 and putatively with acyl chains of phosphatidylcholine lipids bound to RyR1 (Figure 1B).

The second simvastatin binding site Sim-2 is located near the N-terminal region of the S6 helix close to the luminal TMD surface, where simvastatin interacts with residues L4909, Y4910, and V4913 (Figure 1C). Compared to Sim-1, the simvastatin binding pose in Sim-2 is less engaged, exhibiting viewer contacts with RyR1. The density signal for simvastatin is less strong in Sim-2 than in Sim-1, implying lower occupation of Sim-2. Moreover, superposition of Sim-2 with the TMx-containing apo RyR1 structure revealed steric overlaps of the 2,2-dimethylbutyrate ester group and the lactone group of simvastatin with the N-terminal TMx helix backbone at residues 4,324–4,325 and L4320 near the luminal TMD surface, respectively (Supplemental Figure 8). Hence, by occupying Sim-2, simvastatin would sterically displace TMx or require an induced fit, altering its binding pose in the presence of TMx.

Interestingly, the amino acid residues interacting with simvastatin in Sim-1 and Sim-2 are highly conserved across mammalian species and RyR isoforms, as shown by multiple sequence alignments (Supplemental Figure 9A). Structural alignments of Sim-1 (Supplemental Figure 9B) and Sim-2 (Supplemental Figure 9C) in mouse RyR1 with human RyR2 indicate that the positioning of the residue side chains interacting with simvastatin is also highly conserved for both binding sites. Hence, RyR2 and RyR3 are predicted to interact with simvastatin similar to RyR1.

Simvastatin lactone differs from the acid form due to the closed lactone ring that can modify interaction specificities (Supplemental Figure 1), e.g., the closed lactone ring impairs binding to the HMG-CoA reductase (4). However, the lactone group of simvastatin does not appear to determine the occupation of Sim-1 or Sim-2 on RyR1. In the RyR1-simvastatin structure, the acid form of simvastatin could likely adopt binding poses at Sim-1 and Sim-2 similar to the lactone form without steric clashes, suggesting that simvastatin binds to RyR1 independent of the lactone ring hydrolysis. Accordingly, simvastatin binding to RyR1 has been previously reported for both the lactone and acid forms (14).

**Figure 3:**
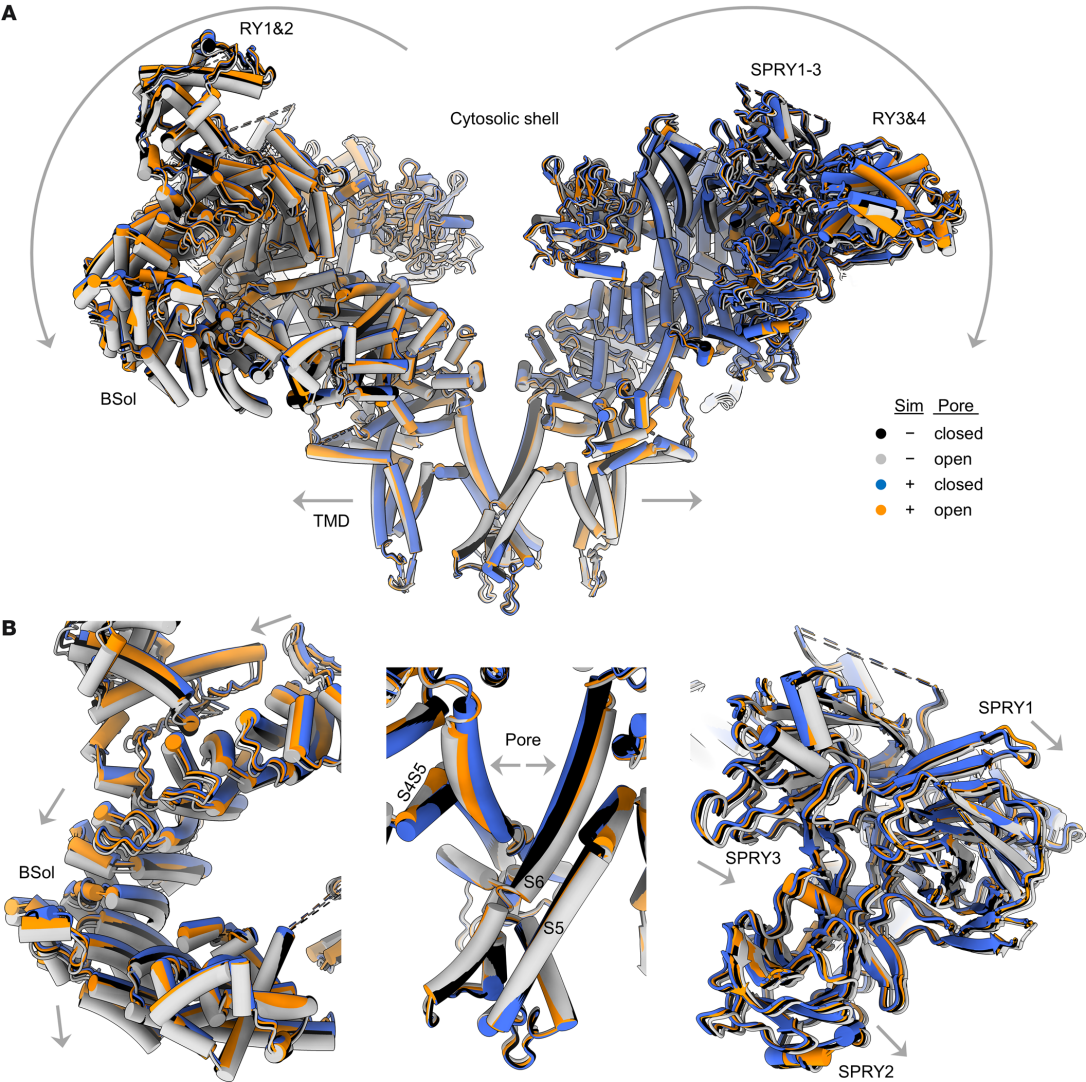
RyR1 pore gating independent of cytosolic shell motion in the presence of simvastatin. (**A** and **B**) Structural comparison of RyR1 with and without simvastatin showing closed and open conformational states of the pore. (**A**) Side view on 2 opposing protomers of (homotetrameric) RyR1 shown as a cartoon model (α-helices depicted as cylinders and β-sheets as arrows). (**B**) Magnifications showing the BSol domain (left) and SPRY1-3 region (right) of the cytosolic shell and the inner pore of TMD (center). In the absence of simvastatin, the opening of the RyR1 pore is accompanied by downward and outward motions (arrows) of the regulatory cytosolic shell including BSol and SPRY1-3 (closed model in black; open model in gray). In the presence of simvastatin, the RyR1 pore opens while the cytosolic shell remains in a closed-like conformation (closed model in blue, open model in orange).

Comparisons of RyR1 structures with and without simvastatin did not reveal significant structural alterations in the closed or open conformation of the pore. However, the cytosolic shell of RyR1 remained in a closed-like conformation upon pore opening promoted by simvastatin (Figure 3A). In the open RyR1 structure with simvastatin, the BSol and SPRY domains of the cytosolic shell did not show the downward and outward motions typical for the conformational transition into the open state (Figure 3B). Hence, simvastatin binding to the pore region may facilitate direct pore opening events independent of the cytosolic shell conformation and its regulatory function.

### In vitro activation of isolated RyR1 channels by simvastatin in a dose-dependent manner.

To functionally characterize simvastatin binding on RyR1 in vitro, microsomal RyR1 was prepared from mouse skeletal muscle and subjected to radioligand binding assays or single-channel recordings while assessing simvastatin dose responses.

**Figure 4:**
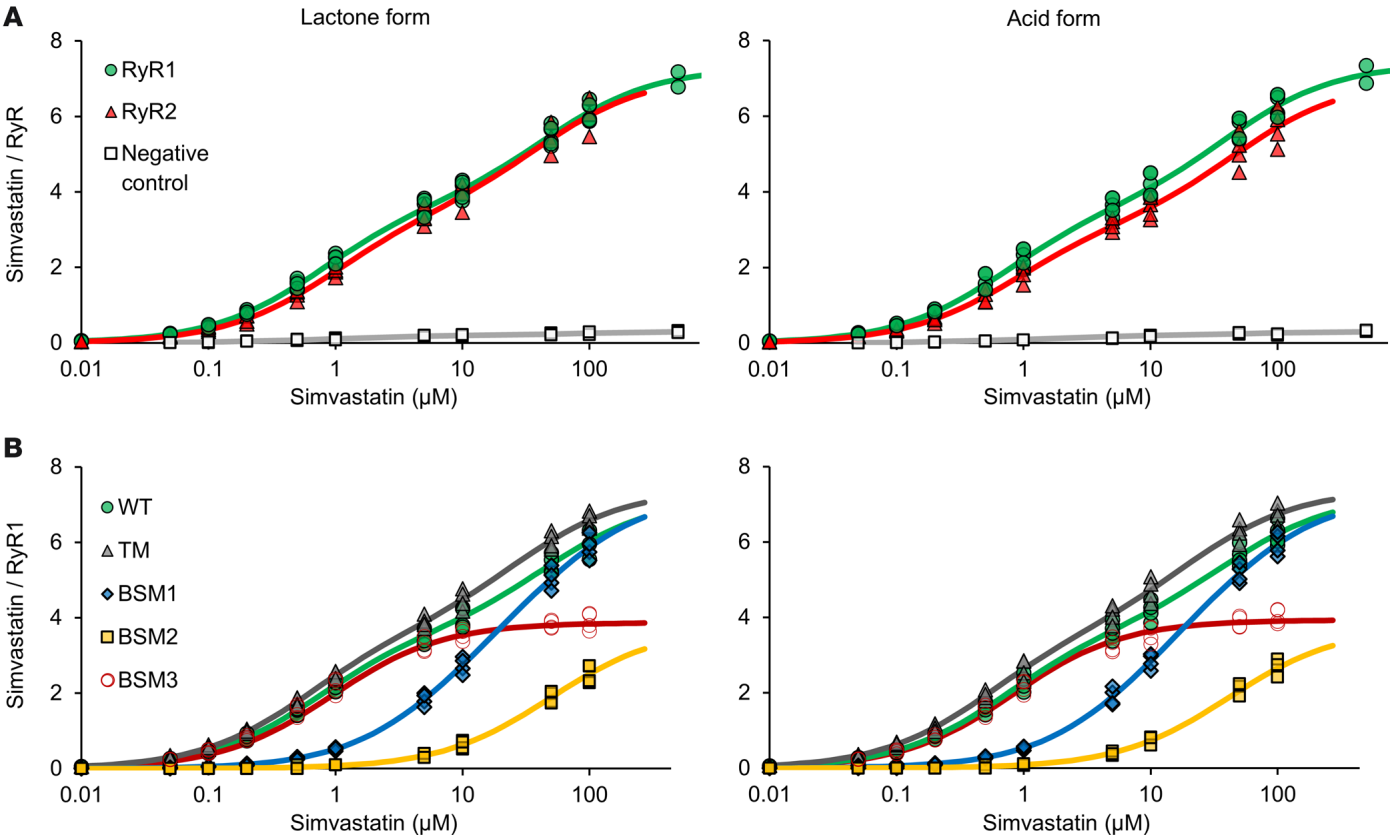
Simvastatin dose response of microsomal RyR channels in radioligand binding assays. (**A**) Radiolabeled simvastatin binding to endogenous RyR1 (green) and RyR2 (red) channels on SR microsomes prepared from mouse skeletal and cardiac muscle tissues, respectively. Microsomal preparations from untransfected HEK293 cells (without RyR) served as negative control (white). (**B**) Radiolabeled simvastatin binding to recombinant WT RyR1 (green), TM mutant (gray), and Sim-1/Sim-2 binding site mutants (BSM1: W4792A in blue; BSM2: Y4789A+W4792A in yellow; BSM3: Y4910A in dark red) on ER microsomes prepared from transiently transfected HEK293 cells. The binding curves of simvastatin lactone (left) or simvastatin acid (right) were analyzed using a model-fitting function for 2 specific binding sites. Estimated *K_D_* and B_max_ values of the 2 simvastatin binding sites are listed in Table 1.

In the radioligand binding assays, RyR1 was incubated with radiolabeled simvastatin lactone at concentrations ranging from 0.01 to 500 μM (Figure 4, A and B). The corresponding dose-response curve indicated initial simvastatin binding to RyR1 starting at submicromolar simvastatin concentrations. Increasing the simvastatin concentration to over ~10 μM resulted in more than 4 bound simvastatin molecules per tetrameric RyR1, indicating a second binding site on RyR1 protomers. The dose-response curve did not reach saturation at the highest simvastatin concentration of 500 μM in the radioligand binding assays (given limitations in the availability of radiolabeled material as well as in the solubility of simvastatin), implying low affinity of the second simvastatin binding site. The dose-response curve was accurately fitted by considering 2 specific binding sites (*R*^2^ = ~0.99). The estimated dissociation constants (*K_D_*) for simvastatin lactone bound to RyR1 are 0.74 ± 0.06 μM (at the higher-affinity site) and 46.6 ± 7.7 μM (at the lower-affinity site), while the corresponding maximum ligand site occupancy (B_max_) values are 3.63 ± 0.15 and 3.60 ± 0.15, respectively (Table 1). The higher- and lower-affinity simvastatin binding sites may correspond to Sim-1 and Sim-2 in the RyR1-simvastatin structure, respectively, since Sim-1 showed stronger occupancy than Sim-2 (Figure 1). Interestingly, the RyR2 isoform prepared from mouse hearts showed a simvastatin dose-response curve in the radioligand binding assays similar to RyR1 (Figure 4A), hence agreeing with the high conservation of Sim-1 and Sim-2 binding sites between RyR1 and RyR2 (Supplemental Figure 9). Similar dose-response curves were obtained for simvastatin in its acid form (Figure 4A), implying that the simvastatin binding to RyR1 or RyR2 is not substantially affected by the hydrolysis of the lactone group.

To further validate simvastatin binding on RyR1 channels, recombinant RyR1 on ER microsomes prepared from transiently transfected HEK293 cells was subjected to radioligand binding assays (Figure 4B). ER microsomes from untransfected HEK293 cells (negative control without RyR1) did not show significant nonspecific background binding of simvastatin (Figure 4A). For recombinant WT RyR1, we detected similar simvastatin dose-response curves as for endogenous RyR1 from skeletal muscle (Figure 4, A and B). Compared with WT, the TM mutation of RyR1 shifted the simvastatin dose-response curves to minimally higher binding affinities for both the lactone form (estimated *K_D1_*: 0.58 ± 0.09 μM for TM versus 0.70 ± 0.09 μM for WT) and the acid form (estimated *K_D1_*: 0.50 ± 0.08 μM for TM versus 0.69 ± 0.09 μM for WT), suggesting that the TM mutation does not substantially affect simvastatin binding (Figure 4B and Table 1). To confirm simvastatin binding specificity, we introduced binding site mutations (BSMs) of Sim-1, a single mutant W4792A (BSM1) and a double mutant Y4789A + W4792A (BSM2), and Sim-2, Y4910A (BSM3). Simvastatin binding to the higher-affinity site was strongly decreased by BSM1 (*K_D1_*: ~7.1 μM for lactone and acid forms) and nearly completely prevented by BSM2 (*K_D1_*: not detectable), thus confirming Sim-1 as the higher-affinity simvastatin binding site (Figure 4B and Table 1). BSM3 did not significantly impair simvastatin binding to the higher-affinity site (*K_D1_* for BSM3 remained similar to WT) but to the lower-affinity site (*K_D2_*: not detectable), thus confirming Sim-2 as the lower-affinity simvastatin binding site (Figure 4B and Table 1).

**Figure 5:**
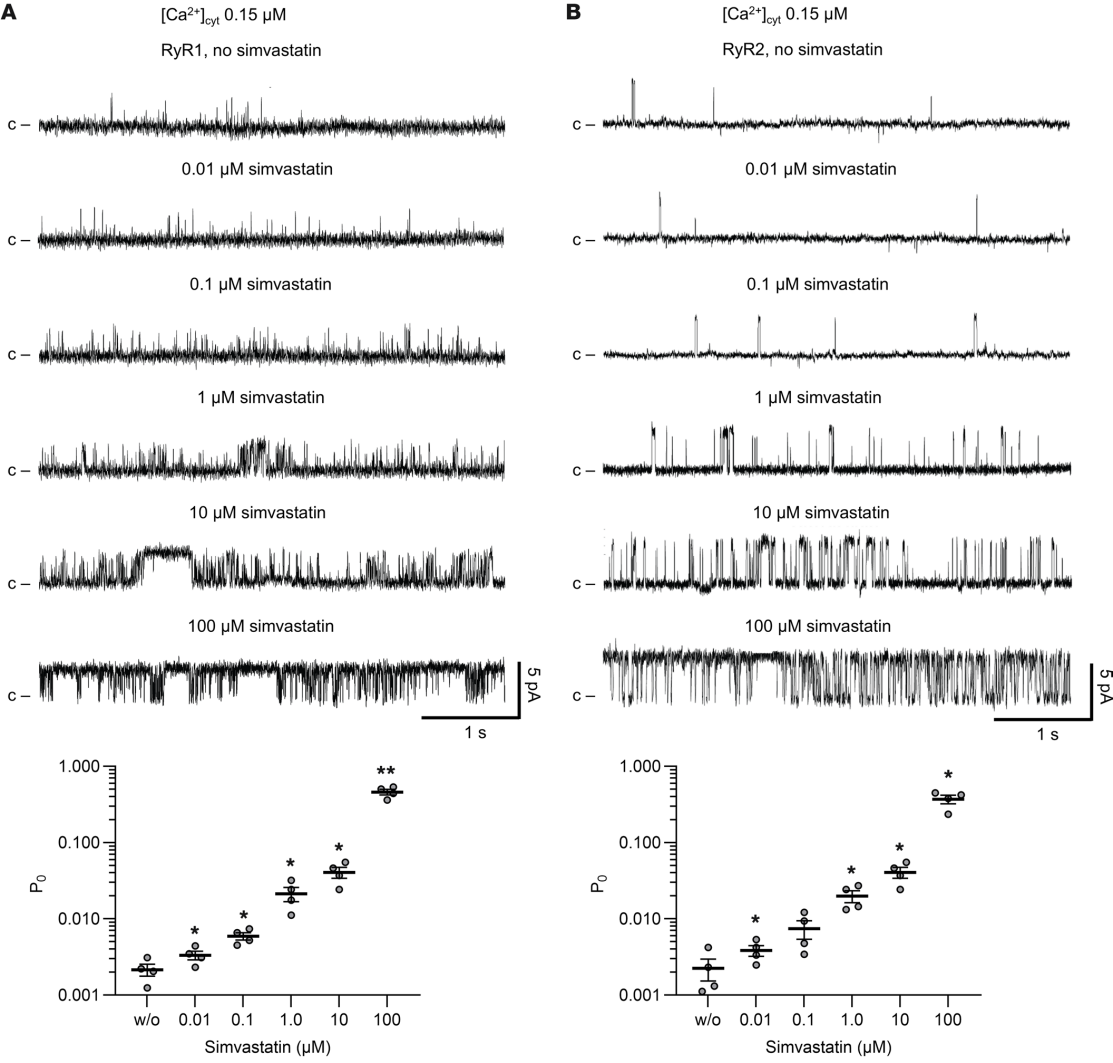
Single-channel recordings of RyR1 or RyR2 treated with simvastatin. (**A**) Microsomal RyR1 or (**B**) RyR2 channels were prepared from mouse skeletal muscle or cardiac muscle tissues, respectively, and reconstituted in planar lipid bilayers. Current traces (top) were recorded at 150 nM free Ca^2+^ without or with simvastatin (acid form) at concentrations as indicated and the corresponding open probabilities (bottom) quantified (*N* = 4). Single-channel traces shown are from the same RyR1 and RyR2 channels treated with increasing simvastatin concentrations (from 0.01 to 100 μM). Channel openings in current traces are represented as upward deflections, while baseline currents correspond to the closed state (c) of RyR. Upon addition of simvastatin at submicromolar concentrations, the open probability (P_O_) of RyR1 significantly increased (reaching an ~1.5-fold increase at 0.01 μM and an ~10-fold increase at 1 μM simvastatin) and continued to increase at micromolar concentrations (~214-fold increase at 100 μM simvastatin). RyR2 showed a simvastatin dose response similar to RyR1. Data are expressed as mean ± SEM. Two-tailed Student’s *t* test; *P < 0.05, **P < 0.01.

To confirm activation of RyR1 by simvastatin, we reconstituted microsomal RyR1 channels in planar lipid bilayers. Following baseline recordings of single RyR1 channels at 150 nM free Ca^2+^, the dose response of simvastatin (acid form) on RyR1 was measured by increasing the simvastatin concentration sequentially in four 10-fold steps from 0.01 to 100 μM. Simvastatin significantly increased the RyR1 open probability from 0.2% at baseline to 2.1% at 1 μM simvastatin and 4.1% at 10 μM simvastatin; a further strong increase to 46% open probability was reached at 100 μM simvastatin (Figure 5A). Hence, substantial activation of single RyR1 channels in planar lipid bilayers required micromolar simvastatin concentrations under in vitro experimental conditions. Our results are in accordance with previously reported single-channel recordings showing a similar dose response of RyR1 to simvastatin at micromolar concentrations (14).

Interestingly, the simvastatin dose responses of RyR1 and RyR2 in planar lipid bilayers resembled each other (Figure 5B). Similar to RyR1, we observed only simvastatin-induced activation but no inhibition of RyR2. Hence, our results did not confirm the previously reported inhibition of RyR2 at low micromolar concentrations of simvastatin (14).

To analyze the functional effects of the Sim-1 and Sim-2 binding site mutations, we subjected BSM2 and BSM3 to [^3^H]ryanodine binding assays. Ryanodine preferentially binds to the open state of RyR1. Hence, an increase or reduction in [^3^H]ryanodine binding is linked to functional activation or deactivation of RyR1, respectively (14, 35). For BSM2 and BSM3, the level of bound [^3^H]ryanodine did not significantly change compared with recombinant WT RyR1 at 0.3 or 10 μM free Ca^2+^ (Supplemental Figure 10A), implying that these simvastatin binding site mutations did not affect RyR1 activity in the absence of simvastatin.

Addition of simvastatin acid to WT RyR1 increased [^3^H]ryanodine binding in a dose-dependent manner (Supplemental Figure 10B). At 0.3 μM free Ca^2+^, the corresponding dose-response curve exhibited a sigmoidal shape with an EC_50_ value of 4.99 ± 0.89 μM and a maximum fold change (E_max_) of 1.75 ± 0.02 in bound [^3^H]ryanodine. At 10 μM free Ca^2+^, the EC_50_ value shifted to an ~7-fold lower simvastatin concentration of 0.73 ± 0.09 μM and the E_max_ value increased by ~19% to 2.08 ± 0.02, implying that the simvastatin-induced RyR1 activation is promoted by Ca^2+^. The latter EC_50_ value is in line with the *K_D1_* value of the Sim-1 site in WT RyR1 (Table 1).

Both BSM2 (at Sim-1) and BSM3 (at Sim-2) substantially reduced the dose response to simvastatin in the [^3^H]ryanodine binding assays at 0.3 or 10 μM free Ca^2+^ (Supplemental Figure 10B). BSM2 resulted in increased EC_50_ and reduced E_max_ values (EC_50_: ~7.15 ± 0.96 μM; E_max_: 1.86 ± 0.02 at 10 μM free Ca^2+^), whereas BSM3 showed unchanged EC_50_ but reduced E_max_ values (E_max_: 1.32 ± 0.01 at 10 μM free Ca^2+^) compared with WT. The reduction of E_max_ was more pronounced by BSM3 than by BSM2.

Taken together, these results suggest that simvastatin-induced RyR1 activation is predominantly mediated by the Sim-1 site at submicromolar and low micromolar simvastatin concentrations and by the Sim-2 site at higher micromolar simvastatin concentrations.

### Enhanced statin-induced myopathy of RyR1-TM mice is mitigated by treatment with the Rycal drug S107.

To assess side effects of simvastatin on skeletal muscles in vivo, we treated WT mice (C57BL/6J) with ~50 mg/kg/day simvastatin (acid form; *N* = 10) or placebo (solvent without simvastatin; *N* = 10) administered for ~6 weeks in drinking water. In an additional test group, the Rycal drug S107 (~50 mg/kg/day) was added to the former drinking water to stabilize (leaky) RyR channels in the presence of simvastatin (*N* = 10). Moreover, 1 WT test group (*N* = 5) was treated with a 2.5-fold lower simvastatin dose (~20 mg/kg/day) for ~8 weeks. In vivo tests (including grip strength) and blood tests (including blood gases, ions, and creatine kinase [CK] levels) showed no significant differences between these test groups after treatment (Supplemental Figures 11 and 12, and Supplemental Table 2). After euthanasia, the specific force productions were measured ex vivo for the extensor digitorum longus (EDL), soleus, and diaphragm muscles at electrical stimulation frequencies ranging from 1 to 120 Hz. The force frequency relationship plots exhibited typical sigmoid curves with initial muscle activation at lower frequencies and maximal isometric force output at higher frequencies (Supplemental Figure 13).

**Figure 6:**
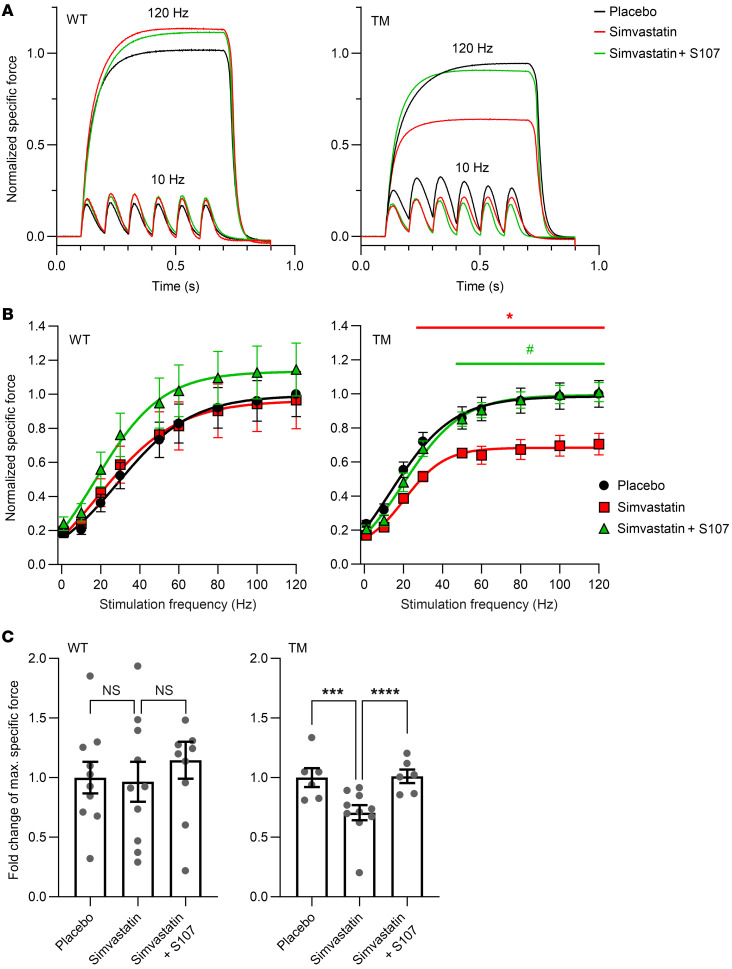
Effect of simvastatin treatment on the force-frequency relationship of the diaphragm muscle dissected from WT versus heterozygous TM mice. (**A**–**C**) WT or heterozygous TM mice were treated with placebo (*N* = 10 WT/6 TM), ~50 mg/kg/day simvastatin (*N* = 10 WT/10 TM), or the latter together with ~50 mg/kg/day S107 (*N* = 10 WT/6 TM) for 6 weeks (WT)/4 weeks (TM). Representative specific force traces measured ex vivo at 10 or 120 Hz (**A**), force-frequency relationships (**B**), and fold changes (**C**) of the maximal specific force are shown for the diaphragm dissected from WT (left) or TM (right) mice. Data are expressed as mean ± SEM. In WT (left), simvastatin treatment did not significantly affect the diaphragmatic force production. In TM (right), simvastatin treatment induced a significant diaphragmatic force reduction at ≥20 Hz compared with placebo (2-way ANOVA, *P < 0.05, ***P < 0.001), while cotreatment with Rycal S107 resulted in a significant force recovery at ≥50 Hz (2-way ANOVA, #*P* < 0.05, ****P < 0.0001). At 120 Hz, the loss in TM diaphragmatic force production due to simvastatin treatment reached ~29%.

Simvastatin treatment (~50 mg/kg/day) of WT mice resulted in significantly improved maximal specific forces of the EDL (~22% increase at 120 Hz; *P* < 0.001; Supplemental Figure 13A) and soleus muscles (~25% increase at 120 Hz; *P* < 0.05; Supplemental Figure 13B), while the diaphragm force production remained similar to placebo (Figure 6). Similarly, treatment with the 2.5-fold lower simvastatin dose (~20 mg/kg/day) showed a trend for improved EDL force production, but to a more modest extent (~13% increase at 120 Hz; not significant; Supplemental Figure 13A). Importantly, we did not detect adverse simvastatin side effects on skeletal muscles in WT mice.

Simvastatin-induced specific force improvement was not affected by the cotreatment with S107 in EDL (Supplemental Figure 13A), whereas S107 showed a trend to partially prevent the increase in the soleus (Supplemental Figure 13B). The diaphragm-specific force was unaltered by the S107 cotreatment (Figure 6, B and C). Hence, S107 had no significant impact on the simvastatin treatment–induced effects on ex vivo muscle functions of EDL, soleus, and diaphragm compared with simvastatin alone in WT.

Previous reports suggested that pathogenic mutations can render RyR1 channels more susceptible to statin-induced activation in skeletal muscle (26, 27). To investigate if the TM mutation promotes simvastatin side effects in skeletal muscle, we treated heterozygous TM mice with placebo (*N* = 6), simvastatin (~50 mg/kg/day; *N* = 10), or simvastatin/S107 (both ~50 mg/kg/day; *N* = 6) for 4 weeks in the same manner as for WT. Similarly to WT mice, simvastatin treatment of heterozygous TM mice did not significantly alter in vivo behavioral test results (including grip strength) or CK blood levels (Supplemental Figures 11 and 14). However, in contrast with WT mice, simvastatin-treated heterozygous TM mice did not show improved specific force production, but, instead, a trend for reduced specific force production in the EDL and soleus muscles ex vivo compared with placebo (Supplemental Figure 15). In the diaphragm ex vivo, simvastatin treatment of heterozygous TM mice resulted in a significantly reduced peak specific force production (~29% decrease at 120 Hz; *P* < 0.001), while cotreatment with S107 completely prevented this reduction in peak specific force production (Figure 6). During repetitive muscular contractions, simvastatin did not significantly alter the time-dependent decay (or fatigue) in specific force production of the EDL, soleus, or diaphragm in WT or heterozygous TM mice (Supplemental Figure 16).

Taken together, these results demonstrate that the TM mutation aggravates simvastatin side effects in skeletal muscle and that these effects are mitigated by the (RyR-stabilizing) Rycal drug S107, implying a crucial role of RyR1 in mediating SAMS.

## Discussion

The discovery of the LDL receptors by Brown and Goldstein (37, 38) and the development of statin drugs (initially compactin, alias mevastatin, by Akiro Endo at Sankyo in 1972 and mevinolin, alias lovastatin, by Alfred Albert at Merck under the direction of Roy Vagelos in 1979) are major accomplishments in the development of effective therapeutics for heart disease (1–3). SAMS are major side effects limiting the use of statins (6, 7, 10). The present study sheds light on the previously perplexing question: What are the causes of SAMS? Addressing this question could potentially lead to development of statins that do not bind to RyR1 and are better tolerated by patients who need to take them.

We structurally and functionally characterized the side effects of simvastatin in skeletal muscle associated with SAMS based on its binding to RyR1. Previous reports showed how statins interact with HMG-CoA reductase, the pharmacological target of statin therapies (4). As the mechanism of action to downregulate cholesterol biosynthesis, statin drugs inhibit the HMG-CoA reductase by sterically blocking access to the HMG-CoA substrate binding pocket. Accordingly, x-ray crystal structures revealed that the hydrophilic dihydroxyheptanoic acid group (opened lactone ring), common to all statins, intrudes deep into the active site of the enzyme by adopting an HMG-like conformation in the HMG-CoA binding pocket, while the bulky hydrophobic moiety occupies a shallow nonpolar groove partially blocking the binding surface for CoA (Supplemental Figure 17A) (4). Despite their structural diversity, different statin drugs such as simvastatin and atorvastatin exhibit similar complementary interaction surfaces in the substrate binding pocket of the HMG-CoA reductase, explaining their shared mechanism of action (4).

Our cryo-EM analyses identified 2 simvastatin binding sites per protomer in the pore-forming TMD of homotetrameric RyR1 (Figure 1) that both substantially differ from the statin HMG-CoA reductase binding site. Compared with HMG-CoA reductase, the simvastatin binding sites Sim-1 and Sim-2 of RyR1 do not form a tight binding pocket for the hydrophilic dihydroxyheptanoic acid group (opened lactone ring) of statins (Supplemental Figure 17, A and B). Instead, the simvastatin binding at the Sim-1 and Sim-2 sites is dominated by hydrophobic interactions (largely mediated by the hexahydronaphthalene core moiety and the 2,2-dimethylbutyrate ester group of simvastatin).

The Sim-1 site is characterized by a hydrophobic pocket between the transmembrane helices S3 and S4, which is exposed upon steric displacement of the W4792 side chain. The 2,2-dimethylbutyrate ester group of simvastatin protrudes into this pocket, engaging in tight hydrophobic interactions with Sim-1 (Figure 2).

Sim-2 is formed by the hydrophobic N-terminal S6 region located near the luminal site. At Sim-2, simvastatin exhibits a less engaged binding pose, resulting in a lower occupancy compared with Sim1. By occupying Sim-2, simvastatin would sterically clash with the auxiliary transmembrane helix TMx that is absent in the RyR1-simvastatin structures (Supplemental Figure 8), as well as in most of the previously reported RyR1 structures without simvastatin (33). Using radiolabeled simvastatin in binding assays on microsomal RyR1 (not solubilized), we confirmed the existence of a second low-affinity simvastatin binding site in the absence of detergents (Figure 4). Moreover, the TMx stability in vivo is unknown. Plausible mechanisms to explain Sim-2 occupancy could either involve the steric displacement of TMx by simvastatin or an induced fit of its binding pose in the presence of TMx putatively stabilized in the lipid bilayer environment.

Both Sim-1 and Sim-2 are highly conserved across mammalian RyR isoforms (Supplemental Figure 9), resulting in nearly identical binding curves detected for simvastatin to RyR1 and RyR2 (Figure 4A). However, simvastatin (1 μM) has been reported to activate RyR1 but inhibit RyR2 (14). These opposite RyR isoform–dependent responses to statin might contribute to the previously reported discrepancy of adverse versus beneficial effects on skeletal (RyR1) and cardiac muscle (RyR2) in statin therapies, respectively (2, 16, 17). Given the high conservation of the Sim-1 and Sim-2 sites, opposite statin effects on RyR1 versus RyR2 activities might be caused by isoform-dependent differences in the signal transduction (from Sim-1/Sim-2 to the inner pore gate) but not directly in Sim-1 or Sim-2. However, we did not detect any conformational changes in the pore region of RyR1 upon simvastatin binding that could explain differences in signal transduction. Moreover, the simvastatin-induced initial inhibition of RyR2 was reportedly reversed by higher micromolar simvastatin concentrations (14); hence, occupying Sim-1 and Sim-2 may inhibit and activate RyR2 in a dose-dependent fashion, respectively. Interestingly, our single-channel recordings did not show inhibition but only activation of RyR2 induced by simvastatin initially at submicromolar concentrations, hence resembling RyR1 (Figure 5). This apparent discrepancy compared with the previous study (14) might be explained by the low free Ca^2+^ condition (0.15 μM instead of 10 μM) in our single-channel recordings resulting in less activated RyR2 at baseline.

Importantly, simvastatin belongs to the type 1 statin drugs that share a hexahydronaphthalene ring system as a core structure with the lactone functional group attached to it (13). Besides simvastatin, the type 1 statin subgroup comprises lovastatin, mevastatin (compactin), and pravastatin (Supplemental Figure 1). Given their structural similarities, type 1 statin drugs are expected to exhibit comparable interactions with RyR1 at the Sim-1 and Sim-2 sites. Indeed, similar dose responses have been shown for simvastatin and pravastatin in [^3^H]ryanodine binding assays of RyR1 (29), implying that the substitution of 2,2-dimethylbutyrate (specific for simvastatin) with the slightly smaller 2-methylbutyrate ester moiety (typical for type 1 statins) does not disturb the interaction with Sim-1 or Sim-2.

Statins of the structurally more diverse type 2 subgroup (e.g., atorvastatin and rosuvastatin) have also been reported to stimulate RyR1 similar to type 1 statins (Supplemental Figure 1), suggesting that type 1 and 2 statins share a similar mechanism of action on RyR1 (29). Compared with type 1 statins, type 2 statins typically contain a pyrrole- or pyrimidine-based ring system larger than naphthalene along with a 4-fluorophenyl group replacing the α-methylbutyrate ester moiety (13). Since type 2 statins share less similarity, their binding modes on RyR1 might be more diverse compared with type 1 statins. Moreover, pathogenic RyR1 mutants/variants might deviate from each other in their statin type–dependent susceptibilities to mediate SAMS. Future studies are warranted to elucidate in detail if type 1 and 2 statin interactions with RyR1 differ mechanistically in their structure-function relationship on RyR1 as well as the impact of (pathogenic) RyR1 mutations and posttranslational modifications on it.

To prevent adverse side effects on RyR1 by type 1 statins, the ester group (at C4) may present a pharmacologically relevant substitution site to design derivatives (Supplemental Figure 1) that lose the ability to mediate tight interactions with the hydrophobic pocket at Sim-1 of RyR1 but keep their inhibitory potential against the HMG-CoA reductase. Interestingly, Hoffman et al. showed that substituting the 2,2-dimethylbutyrate ester group of simvastatin with the more voluminous 2,2-diethylpentanoate did not substantially alter the potency to inhibit the HMG-CoA reductase (IC_50_: 0.9 nM versus 1.9 nM, respectively) (39). Such a steric enlargement of the hydrophobic ester group in type 1 statins could result in steric clashes at Sim-1 on RyR1, hence impairing the interaction. Given the differences in the simvastatin binding modes on RyR1 and HMG-CoA reductase (Supplemental Figure 17), our RyR1-simvastatin structure may provide mechanistic rationale for future therapeutic studies to develop type 1 statin drugs with reduced incidence of SAMS.

The medical relevance of RyR1 as a direct mediator of simvastatin side effects is emphasized by the impact of the TM mutation in RyR1 on simvastatin-treated mice. As shown by our ex vivo muscle-specific force measurements, simvastatin treatment induced significant improvements in the specific forces of the EDL (~22% increase) and soleus muscles (~25% increase) in WT mice, but not in heterozygous TM mice (with rather deteriorating tendencies in the EDL and soleus forces). By contrast, peak diaphragm-specific force production was significantly weakened by simvastatin treatment in heterozygous TM mice (~29% decrease) but not in WT mice (Figure 6 and Supplemental Figures 13 and 15). Under our experimental conditions, WT mice treated with simvastatin did not develop SAMS. This might be similar to the human situation where healthy individuals (without any underlying medical condition or risk factors) would presumably be less likely to develop severe statin intolerance if treated. Taken together, these results suggest that the TM mutation aggravates simvastatin side effects and increases the risk to develop (simvastatin-induced) SAMS.

Interestingly, TM was previously characterized as a recessive pathogenic mutation showing no obvious phenotype in the presence of WT RyR1 (25). However, according to our data, heterozygous carriers of the TM mutation may be predisposed to SAMS. Moreover, it has been reported that the TM mutation does not render RyR1 channels leaky for SR Ca^2+^ under resting conditions but enhances RyR1 sensitivity to activation (19). Therefore, RyR1-activating ligands, including drug-like compounds such as caffeine and statins, may activate TM RyR1 more readily than WT RyR1. Consistent with this, the dominant pathogenic YS mutation of RyR1 was previously described to strongly promote hypermetabolic responses to simvastatin (27). Thus, other pathogenic RyR1 mutations may similarly predispose individuals to SAMS.

In TM, the greater weakness exhibited by the diaphragm in response to simvastatin treatment compared with EDL and soleus suggests the possibility of simvastatin-induced respiratory deficits. In line with this, previous case reports have described statin-induced diaphragmatic weakness in patients presenting with or without elevated CK levels (40, 41). Over time, diaphragmatic weakness may lead to a reduced lung function and respiratory disorders (40, 41).

Importantly, administration of S107 completely prevented simvastatin-induced diaphragmatic weakness in heterozygous TM mice, as shown in ex vivo muscle-specific force measurements. Thus, Rycal drugs like S107 present potential means of preventing or reversing adverse statin side effects on skeletal muscles mediated by RyR1. Moreover, Rycal drugs were established in several studies as potent stabilizers of dysfunctional RyR1 channels linked to congenital myopathies with symptoms similar to SAMS (17, 19, 42). Hence, future studies are warranted to evaluate the efficacy of Rycal drugs in mitigating SAMS.

S107 showed no significant effect on the increase in the maximal specific force production of WT mice treated with simvastatin. Thus, the results for the cotreatment with S107 suggest a key role of RyR1 for the adverse simvastatin side effects on skeletal muscles in heterozygous TM mice but not for the nonadverse simvastatin side effects in WT mice.

As a study limitation, we acknowledge that SAMS exhibit a complex and highly variable clinical presentation (7–10). Hence, it is unlikely that all SAMS share a common underlying pathophysiology. Indeed, besides (dysfunctional) RyR1 channels, several other putative statin off-target effectors have been implicated to contribute to SAMS (13, 15, 16). However, dysfunctional RyR1 channels could contribute to SAMS in diverse fashions (19, 42). Importantly, not only pathogenic mutations but also posttranslational modifications (such as phosphorylation, oxidation, and S-nitrosylation) and protein-protein interactions (such as RyR1-Calstabin, RyR1-Calmodulin, and RyR1-S100A1) can affect RyR1 function (21, 34, 42, 43). Although pathogenic RyR1-related conditions are considered to be amongst the most common causes of neuromuscular diseases, accurate prevalence data are lacking (44, 45). From an unbiased analysis of 130,048 patients admitted to the Geisinger hospital system, pathogenic or likely pathogenic MH susceptibility RYR1 variants were identified in ~1:600 individuals (215/130,048) (45, 46). Moreover, a potential positive correlation between human pathogenic RYR1 variants and SAMS has previously been reported in a small-scale study (47). However, it is not known yet how frequently pathogenic RyR1-related conditions are associated with statin intolerance in patients. Our results from mice imply that not only dominant pathogenic RyR1 mutations like YS (27) but also recessive mutations like TM can predispose heterozygous individuals to SAMS, suggesting a future direction for translational research.

Taken together, the structural, functional, and physiological data provided here suggest a crucial role of dysfunctional RyR1 in contributing to adverse simvastatin side effects in skeletal muscle, making RyR1 a potential therapeutic target to mitigate SAMS.

## Methods

### Sex as a biological variable.

This study examined only male C57BL/6 (WT) mice to reduce experimental variability. It is unknown whether the findings are relevant for female WT mice. For the RyR1-T4706M (TM) knockin mouse model, heterozygous male and female mice were examined. No sex-dependent differences were observed between male and female TM mice regarding simvastatin treatment. Sex was not considered a biological variable in our study.

### Animal models.

The RyR1-T4706M (TM) knockin mouse model has been described previously (25). The 10- to 12-week-old C57BL/6 mice (The Jackson Laboratory) and RyR1-T4706M knockin mice (generated in-house; age- and gender-matched placebo and treatment groups) were maintained and studied according to protocols approved by the Institutional Animal Care and Use Committee of Columbia University (reference no. AC-AACF4750) and the University Committee on Animal Resources of the University of Rochester (reference no. UCAR 2006-114E). Animals were randomly assigned to one of the designed groups. All in vivo animal experiments were performed by investigators blinded to genotype and treatment groups.

### Treatment of mice with simvastatin and S107.

Mice were fed chow diet and housed in a barrier facility with 12-hour/12-hour light/dark cycles. The statin drug simvastatin (~20 or ~50 mg/kg/day) and Rycal drug S107 (~50 mg/kg/day) were administered ad libitum as described previously (48, 49). In brief, simvastatin lactone powder (Thermo Fisher Scientific) insoluble in water was initially dissolved in ethanol (104 mg/mL simvastatin) and then added to alkaline water (pH > 10), hydrolyzing simvastatin lactone into its water-soluble acid form (final: 0.167 or 0.417 mg/mL simvastatin). The alkaline water was neutralized (to pH ~7) with hydrochloric acid. The drinking water for the placebo group was prepared in the same manner (including 0.4% ethanol) but without simvastatin and S107. Drug dosages were calculated with regard to the average water intake (~3 mL/day per mouse) and average mouse body weight (~25 g). Mice were euthanized by CO_2_ overdose followed by cervical dislocation.

### Isometric contractile force assessment of skeletal muscles ex vivo.

The costal diaphragm, EDL, and soleus muscles were surgically excised from euthanized WT or heterozygous TM mice. Isometric contractile properties and fatigue characteristics were determined as described previously (43, 48). Ex vivo muscles were mounted into jacketed tissue bath chambers and continuously perfused with oxygenated Krebs solution at 28°C. Square wave pulses were applied to achieve supramaximal stimulation (model S407A; Aurora, Grass Instruments). The force-frequency relationship was assessed by sequentially stimulating the muscles for 0.6 seconds at 10, 20, 30, 50, 60, 80, 100, and 120 Hz with 1 minute between each stimulation train. Muscle fatigue was measured as the loss in specific force production in response to repetitive electrical stimulation (30 Hz, 0.3-second duration) over the course of 5 minutes. After measurement of the optimal muscle length Lo (at which maximal isometric tension is produced), muscles were dried and weighed. The muscle force production was normalized to the total muscle strip cross-sectional area (units in N/cm^2^) determined by dividing muscle mass by its length and tissue density (1.056 g/cm^3^).

### Grip strength assessment.

The forelimb grip strength was measured using a grip strength meter (GPM-100; Melquest) as previously described (48). To record the peak pull force, a mouse was allowed to grasp the bar mounted on the force gauge and connected to a digital force transducer.

### Hanging task.

The sustained grip strength was assessed by a wire hanging test. Mice were hung from an inverted metal cage. The time to fall was determined for each mouse in 3 consecutive trials with 5 minutes of rest between trials. The maximum duration per trial was set to 60 seconds.

### Serum CK level.

Following euthanasia as described above, blood was collected from the mouse via cardiac puncture and put in a EDTA-coated collection tube (BD Biosciences) to prevent clotting. Serum CK levels were assessed using a creatine kinase activity assay kit (MAK116, (Sigma-Aldrich) according to the manufacturer’s instruction.

### Purification of endogenous RyR1 from mouse skeletal muscle.

Endogenous RyR1 was purified from mouse skeletal muscle as previously described (34). For all purification steps, buffers were kept ice-cold unless otherwise stated. Skeletal muscle tissue was dissected from the back and thigh of 8- to 20-week-old C57BL6 mice. Approximately 30 g of skeletal muscle tissue was lysed in ~200 mL buffer A [10 mM Tris maleate, pH 6.8, 1 mM EGTA, 1 mM benzamidine hydrochloride, and 0.5 mM 4-(2-aminoethyl)benzenesulfonyl fluoride hydrochloride (AEBSF)] using a Waring blender. To remove debris, the skeletal muscle lysate was centrifuged for 10 minutes at 11,000*g*. The supernatant was filtered through cheesecloth. Membrane fractions were pelleted by centrifugation at 36,000*g* for 30 minutes and solubilized in buffer B (10 mM HEPES, pH 7.4, 0.8 M NaCl, 1% CHAPS, 0.1% phosphatidylcholine, 1 mM EGTA, 2 mM DTT, 0.5 mM AEBSF, 1 mM benzamidine hydrochloride, and 1 protease inhibitor tablet [Pierce]) using a glass tissue grinder (Kontes, MilliporeSigma) for homogenization. The membrane suspension was diluted with buffer C (same as buffer B but without NaCl) at a 1:1 ratio, and the homogenization was repeated. To remove CHAPS-insoluble material, solubilized membrane proteins were centrifuged at 100,000*g* for 30 minutes, and the supernatant was filtered and loaded onto a HiTrap Q HP column (5 mL; GE Healthcare) previously equilibrated with buffer D [10 mM HEPES, pH 7.4, 400 mM NaCl, 0.25% CHAPS, 1 mM EGTA, 0.5 mM Tris(2-carboxyethyl)phosphine hydrochloride), and 0.01% 1,2-dioleoyl-*sn*-glycero-3-phosphocholine (Avanti)]. The immobilized protein on the HiTrap Q HP column was washed with 6 column volumes of buffer D and then eluted using a linear gradient from 480 to 550 mM NaCl with buffers D and E (same as buffer D but with 600 mM NaCl). RyR1-containing fractions were pooled and concentrated to ~0.4 mL using 100,000 kDa cutoff centrifugation filters (MilliporeSigma). Finally, RyR1 was purified by size-exclusion chromatography using a TSKgel G4SWXL column (Tosoh Bioscience) with buffer F (same as buffer D but with 150 mM NaCl). RyR1 fractions were pooled and concentrated to >10 g/L using 100,000 kDa cutoff centrifugation filters (MilliporeSigma).

For cryo-EM, samples of RyR1 (final: ~15 μM RyR1-protomer) were prepared with 5 mM EGTA only or with 10 mM Na-ATP, 5 mM caffeine, and 30 μM free Ca^2+^ and incubated with 10 mM simvastatin lactone dissolved in DMSO (final: ~1%) for at least 20 minutes on ice. Free Ca^2+^ concentrations were calculated using MaxChelator (https://somapp.ucdmc.ucdavis.edu/pharmacology/bers/maxchelator/webmaxc/webmaxcE.htm).

### Cryo-EM data collection, processing, and model building.

Cryo-EM grids were prepared with a Vitrobot Mark IV (Thermo Fisher Scientific) used as a vitrification device as described previously (34, 35, 50). In brief, UltrAuFoil holey gold grids (Quantifoil R 0.6/1.0, Au 300) were cleaned with EasiGlow (PELCO). Three-microliter samples of purified proteins were applied per grid. To form thin aqueous layers, grids were blotted (blot force 10 for 8 seconds) with ashless filter paper (Whatman) at 4°C with 100% relative humidity and vitrified by plunge-freezing into liquid ethane chilled by liquid nitrogen. Initial cryo-EM screenings were performed on a Glacios Cryo-TEM (Thermo Fisher Scientific) microscope with a 200 kV extreme field emission gun and a Falcon 3EC direct electron detector (Thermo Fisher Scientific) using EPU software (Thermo Fisher Scientific) to operate the microscope and collect data. High-resolution cryo-EM data were collected on a Titan Krios 300 kV microscope (Thermo Fisher Scientific) equipped with an energy filter (slit width: 20 eV) and a K3 direct electron detector (Gatan). Leginon software was used for automated data acquisition (51). The nominal magnification was ×105,000 in electron counting mode, corresponding to a pixel size of 0.83 Å. The electron dose rate was set to 16 e^−^/pixel/s with 2.5-second exposures for a total dose of 50 to 60 e^−^/Å^2^.

After collection, cryo-EM data were analyzed in cryoSPARC (52) using patch motion correction and patch CTF estimation to align image stacks and estimate defocus values, respectively. RyR1 particles were picked using Topaz trained with preexisting RyR1 templates. After 2 rounds of 2D classification (100 classes), RyR1 particles were pooled from the highest-resolution classes. An ab initio 3D reconstruction of the RyR1 particles was generated. The initial 3D molecular volume of RyR1 was subjected to homogeneous refinement with C4 symmetry imposed followed by heterogeneous refinement with 3 classes to further select the best particles. For RyR1 particles under activating condition, closed and open pore conformations were separated by 3D classification with the small mask focused on the pore region. Particle stacks were symmetry expanded along the C4 axis of RyR1.

For subsequent local refinements, masks were generated as follows: the first mask comprised the N-terminal domain, the 3 SPRY domains, the tandem repeat domain RY1&2, and Calstabin; the second mask comprised the CSol and JSol domains; the third mask comprised the BSol domain; and the fourth mask comprised the pore region including the thumb-and-forefinger, TMD, and C-terminal domains of RyR1. C4 symmetry was imposed in the local refinement of the pore region. Smaller masks focused on the more variable density signals of the S2S3 domain or the tandem repeat domains RY1&2-only and RY3&4-only.

After completing homogeneous and local refinements, composite maps were independently generated for RyR1 particles in closed and open states with or without simvastatin (Supplemental Figure 3). To assemble composite maps, the focused maps from local refinements were aligned to the global consensus map from the corresponding homogeneous refinement and combined in UCSF ChimeraX (53). The pixel size was calibrated using correlation coefficients with a map generated from the crystal structure of the N-terminal domain (54). Atomic models were manually built in Coot (55) starting with a cryo-EM structural model of mouse RyR1 (PDB: 8VJK). Atomic models were refined using real-space refinement in Phenix (56). Figures of RyR1 structures with and without simvastatin were prepared with UCSF ChimeraX (53).

### SR/ER microsome preparation.

SR/ER microsomes from mouse skeletal muscle, heart, or HEK293 cells expressing WT or mutant RyR1 were prepared as previously described (19, 35). In brief, tissue or cell pellets were homogenized on ice using a Teflon-glass homogenizer in buffer H (20 mM Tris maleate, pH 7.4, 1 mM EDTA, 1 mM DTT, and protease inhibitors [Roche]). To remove debris, the homogenate was centrifuged at 4,000*g* for 15 minutes at 4°C. The resulting supernatant was centrifuged at 40,000*g* for 30 minutes or at 50,000*g* for 45 minutes at 4°C for ER or SR microsome preparations, respectively. The pellet was resuspended in storage buffer (10 mM MOPS, pH 7.4, 250 mM sucrose, 1 mM EDTA, 1 mM DTT, and protease inhibitors for ER microsomes; 5 mM PIPES, pH 7.0, 300 mM sucrose, and protease inhibitors for SR microsomes). Aliquots were frozen in liquid nitrogen and stored at –80°C.

### Single-channel data acquisition and analysis of RyR1.

RyR1 channels from SR microsomal preparations of mouse skeletal muscle were reconstituted into planar lipid bilayers for single-channel recordings as described previously (34, 35, 50). In brief, the phospholipids phosphatidylethanolamine and phosphatidylcholine (Avanti Polar Lipids) mixed 3:1 were dissolved in decane (30 mg/mL). The lipid mixture was painted across an aperture (200 μm in diameter) in a polysulfonate cup (Warner Instruments). The planar lipid bilayer formed in the aperture partitioned two 1 mL chambers representing cytoplasmic and SR/ER luminal compartments on the cis and trans sides, respectively. The cis chamber was held at virtual ground, while the trans chamber was connected to the headstage input of a bilayer voltage clamp amplifier (BC-525D; Warner Instruments). The buffers in the 2 chambers were composed as follows: 250/125 mM HEPES/Tris, 50 mM KCl, 1 mM EGTA, and 0.64 mM CaCl_2_, pH 7.35, on cis and 250 mM HEPES, 50 mM KCl, and 53 mM Ca(OH)_2_, pH 7.35, on trans. Free Ca^2+^ concentrations in the cis chamber were calculated using MaxChelator. Microsomal RyR1 channels were added to the cis side of the planar lipid bilayer. To facilitate channel incorporation into the bilayer lipid membrane, the cis side was made hyperosmotic with KCl added up to 400–500 mM. After channel incorporation, the cis chamber was perfused with cis buffer. Single-channel currents of RyR1 were recorded at room temperature before and after adding simvastatin to the cis chamber. The voltage across the planar lipid bilayer was set to 0 mV using the bilayer clamp amplifier. Single-channel currents were filtered at 1 kHz and digitized at 4 kHz. Digidata 1440A and Axoscope 10.2 were used for data acquisition. Clampfit 10.2 (Molecular Devices), GraphPad Prism, and Microsoft Excel 2010 were used for data analysis.

### Radiometric ryanodine and simvastatin binding assays.

Radiometric ryanodine and simvastatin binding assays were conducted on skeletal muscle RyR1, cardiac RyR2, or recombinant RyR1 in microsomal ER/SR preparations as described previously (35). In brief, ~0.1 mg microsomal SR prepared from rabbit heart, skeletal muscle tissue, or HEK293 cells expressing WT or mutant RyR1 were incubated with radiolabeled [^3^H]simvastatin (American Radiolabeled Chemicals) or [^3^H]ryanodine (American Radiolabeled Chemicals) in binding buffer (50 mM Tris-HCl, pH 7.5, 150 mM NaCl, and 25 mM MgCl_2_) at room temperature for 30 minutes. Microsomal preparations from untransfected HEK293 cells (without RyR1) were used as negative control. After incubation, samples were diluted with ice-cold binding buffer and filtered through GF/B Whatman filters. Filters were washed 3 times with 5 mL of wash buffer (10 mM MOPS and 200 mM NaCl, pH 7.4) and dried. The radioactivity retained by the dried filters was quantified by scintillation counting as a measure of [^3^H]ryanodine or [^3^H]simvastatin binding. Nonspecific binding was determined in the presence of 20-fold excess of nonlabeled simvastatin or ryanodine. Data were normalized to saturated [^3^H]ryanodine binding.

### Statistics.

Statistical significance was determined by 2-tailed Student’s *t* test or 2-way ANOVA followed by post hoc tests for multiple comparisons, using the mathematical analysis software GraphPad Prism 10, Microsoft Excel 2010, or R version 4.3. *P* values of less than 0.05 were considered statistically significant. Data in figures are presented as mean ± SEM.

### Study approval.

Mouse studies were approved by the Institutional Animal Care and Use Committee of Columbia University (reference no. AC-AACF4750) and University Committee on Animal Resources of the University of Rochester (reference no. UCAR 2006-114E).

### Data availability.

Numerical values underlying graphical representations in figures are provided in the Supporting Data Values file. Atomic coordinates have been deposited in the PDB and cryo-EM density maps in the Electron Microscopy Data Bank (EMDB). The PDB accession codes are 9NMN, 9NMO, 9NMQ, 9NMP, and 9NMR. The corresponding EMDB accession codes are EMD-49534, EMD-49535, EMD-49536, and EMD-49537.

## Author contributions

GW, HD, and ARM designed research. GW, HD, SR, QY, NZ, LG, JL, YL, CT, JK, and ELF performed research. GW, HD, SR, QY, AC, MCM, AW, RTD, and ARM analyzed data. GW wrote the manuscript. GW, HD, SR, RTD, and ARM edited and revised the manuscript.

## Funding support

This work is the result of NIH funding, in whole or in part, and is subject to the NIH Public Access Policy. Through acceptance of this federal funding, the NIH has been given a right to make the work publicly available in PubMed Central.

- NIH grants R01 DK118240, R01 HL140934, R01 HL178501, R25 HL156002R25, and P01 HL164319 (to ARM).
- NIH grant R01 AR085078 (to HD).
- NIH grant R01 AR078000 (to RTD).

## Supplementary Material

Supplemental data

Supporting data values

## Figures and Tables

**Table 1:**
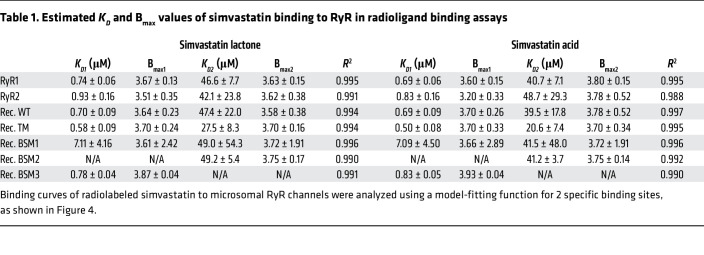
Estimated *K_D_* and B_max_ values of simvastatin binding to RyR in radioligand binding assays

## Version 1 12/15/2025

Electronic publication

## References

[1] Stancu C, Sima A (2001). Statins: mechanism of action and effects. J Cell Mol Med.

[2] Somers T (2023). Statins and cardiomyocyte metabolism, friend or foe?. J Cardiovasc Dev Dis.

[3] Muntean DM (2017). Statin-associated myopathy and the quest for biomarkers: can we effectively predict statin-associated muscle symptoms?. Drug Discov Today.

[4] Istvan ES, Deisenhofer J (2001). Structural mechanism for statin inhibition of HMG-CoA reductase. Science.

[5] Laizure SC (2013). The role of human carboxylesterases in drug metabolism: have we overlooked their importance?. Pharmacotherapy.

[6] Maki KC (2024). Statin-associated muscle symptoms: identification and recommendations for management. Curr Atheroscler Rep.

[7] Ramkumar S (2016). Statin therapy: review of safety and potential side effects. Acta Cardiol Sin.

[8] Alfirevic A (2014). Phenotype standardization for statin-induced myotoxicity. Clin Pharmacol Ther.

[9] Mendes P (2014). Statin-induced rhabdomyolysis: a comprehensive review of case reports. Physiother Can.

[10] Dicken W (2022). Statin associated muscle symptoms: An update and review. Prog Cardiovasc Dis.

[11] Marcoff L, Thompson PD (2007). The role of coenzyme Q10 in statin-associated myopathy: a systematic review. J Am Coll Cardiol.

[12] Liao JK (2002). Isoprenoids as mediators of the biological effects of statins. J Clin Invest.

[13] Patel KK (2022). Molecular targets of statins and their potential side effects: Not all the glitter is gold. Eur J Pharmacol.

[14] Venturi E (2018). Simvastatin activates single skeletal RyR1 channels but exerts more complex regulation of the cardiac RyR2 isoform. Br J Pharmacol.

[15] Schirris TJ (2015). Statin-induced myopathy is associated with mitochondrial complex III inhibition. Cell Metab.

[16] Ward NC (2019). Statin toxicity. Circ Res.

[17] Haseeb M, Thompson PD (2021). The effect of statins on RyR and RyR-associated disease. J Appl Physiol (1985).

[18] Lotteau S (2019). A mechanism for statin-induced susceptibility to myopathy. JACC Basic Transl Sci.

[19] Kushnir A (2020). Intracellular calcium leak as a therapeutic target for RYR1-related myopathies. Acta Neuropathol.

[20] Wehrens XH (2005). Intracellular calcium release and cardiac disease. Annu Rev Physiol.

[21] Betzenhauser MJ, Marks AR (2010). Ryanodine receptor channelopathies. Pflugers Arch.

[22] Sambuughin N (2005). Screening of the entire ryanodine receptor type 1 coding region for sequence variants associated with malignant hyperthermia susceptibility in the north american population. Anesthesiology.

[23] Carsana A (2013). Exercise-induced rhabdomyolysis and stress-induced malignant hyperthermia events, association with malignant hyperthermia susceptibility, and RYR1 gene sequence variations. ScientificWorldJournal.

[24] Jungbluth H (2007). Central core disease. Orphanet J Rare Dis.

[25] Brennan S (2019). Mouse model of severe recessive RYR1-related myopathy. Hum Mol Genet.

[26] Petry NJ (2024). Incidence of statin-associated muscle symptoms in patients taking statins with *RYR1* or *CACNA1S* variants. Per Med.

[27] Knoblauch M (2013). Mice with RyR1 mutation (Y524S) undergo hypermetabolic response to simvastatin. Skelet Muscle.

[28] Iyer KA (2022). Molecular mechanism of the severe MH/CCD mutation Y522S in skeletal ryanodine receptor (RyR1) by cryo-EM. Proc Natl Acad Sci U S A.

[29] Lindsay C (2022). Statin activation of skeletal ryanodine receptors (RyR1) is a class effect but separable from HMG-CoA reductase inhibition. Br J Pharmacol.

[30] Zalk R (2015). Structure of a mammalian ryanodine receptor. Nature.

[31] Yan Z (2015). Structure of the rabbit ryanodine receptor RyR1 at near-atomic resolution. Nature.

[32] Efremov RG (2015). Architecture and conformational switch mechanism of the ryanodine receptor. Nature.

[33] des Georges A (2016). Structural basis for gating and activation of RyR1. Cell.

[34] Weninger G (2024). Structural insights into the regulation of RyR1 by S100A1. Proc Natl Acad Sci U S A.

[35] Melville Z (2022). A drug and ATP binding site in type 1 ryanodine receptor. Structure.

[36] O’Neill ER (2003). Regulation of the calcium release channel from skeletal muscle by suramin and the disulfonated stilbene derivatives DIDS, DBDS, and DNDS. Biophys J.

[37] Brown MS, Goldstein JL (1974). Familial hypercholesterolemia: defective binding of lipoproteins to cultured fibroblasts associated with impaired regulation of 3-hydroxy-3-methylglutaryl coenzyme A reductase activity. Proc Natl Acad Sci U S A.

[38] Brown MS, Goldstein JL (1976). Analysis of a mutant strain of human fibroblasts with a defect in the internalization of receptor-bound low density lipoprotein. Cell.

[39] Hoffman WF (1986). 3-Hydroxy-3-methylglutaryl-coenzyme A reductase inhibitors. 4. Side chain ester derivatives of mevinolin. J Med Chem.

[40] Sulem P (2001). Atorvastatin-induced diaphragmatic muscle impairment. Ann Pharmacother.

[41] Chatham K (2009). Suspected statin-induced respiratory muscle myopathy during long-term inspiratory muscle training in a patient with diaphragmatic paralysis. Phys Ther.

[42] Marks AR (2023). Targeting ryanodine receptors to treat human diseases. J Clin Invest.

[43] Dridi H (2020). Mitochondrial oxidative stress induces leaky ryanodine receptor during mechanical ventilation. Free Radic Biol Med.

[44] Huang K (2021). A systematic review and meta-analysis of the prevalence of congenital myopathy. Front Neurol.

[45] Kelly MA (2021). Leveraging population-based exome screening to impact clinical care: The evolution of variant assessment in the Geisinger MyCode research project. Am J Med Genet C Semin Med Genet.

[46] Yu KD (2024). Evaluation of malignant hyperthermia features in patients with pathogenic or likely pathogenic RYR1 variants disclosed through a population genomic screening program. Anesthesiology.

[47] Isackson PJ (2018). RYR1 and CACNA1S genetic variants identified with statin-associated muscle symptoms. Pharmacogenomics.

[48] Dridi H (2021). Ryanodine receptor remodeling in cardiomyopathy and muscular dystrophy caused by lamin A/C gene mutation. Hum Mol Genet.

[49] Southern WM (2017). Skeletal muscle metabolic adaptations to endurance exercise training are attainable in mice with simvastatin treatment. PLoS One.

[50] Miotto MC (2022). Structural analyses of human ryanodine receptor type 2 channels reveal the mechanisms for sudden cardiac death and treatment. Sci Adv.

[51] Suloway C (2005). Automated molecular microscopy: the new Leginon system. J Struct Biol.

[52] Punjani A (2017). cryoSPARC: algorithms for rapid unsupervised cryo-EM structure determination. Nat Methods.

[53] Goddard TD (2018). UCSF ChimeraX: meeting modern challenges in visualization and analysis. Protein Sci.

[54] Tung CC (2010). The amino-terminal disease hotspot of ryanodine receptors forms a cytoplasmic vestibule. Nature.

[55] Emsley P, Cowtan K (2004). Coot: model-building tools for molecular graphics. Acta Crystallogr D Biol Crystallogr.

[56] Liebschner D (2019). Macromolecular structure determination using X-rays, neutrons and electrons: recent developments in Phenix. Acta Crystallogr D Struct Biol.

