# Supplementary material for: Structural basis for simvastatin-induced skeletal muscle weakness associated with type 1 ryanodine receptor T4709M mutation
Source: J Clin Invest. 2025 Dec 15;135(24):e194490. doi: 10.1172/JCI194490 (PMC12700559; doi:10.1172/JCI194490)
Supplement: Supplemental data [file jci-135-194490-s167.pdf]

Supplemental information for

Structural basis for simvastatin-induced skeletal muscle weakness associated with type 1 ryanodine receptor T4709M mutation

Gunnar Weninger, Haikel Dridi, Steven Reiken, Qi Yuan, Nan Zhao, Linda Groom, Jennifer Leigh, Yang Liu, Carl Tchagou, Jiayi Kang, Alexander Chang, Estefania Luna-Figueroa, Marco C. Miotto, Anetta Wronska, Robert T. Dirksen, Andrew R. Marks

Gunnar Weninger

E-mail: gw2424@cumc.columbia.edu

Andrew R. Marks

E-mail: arm42@cumc.columbia.edu

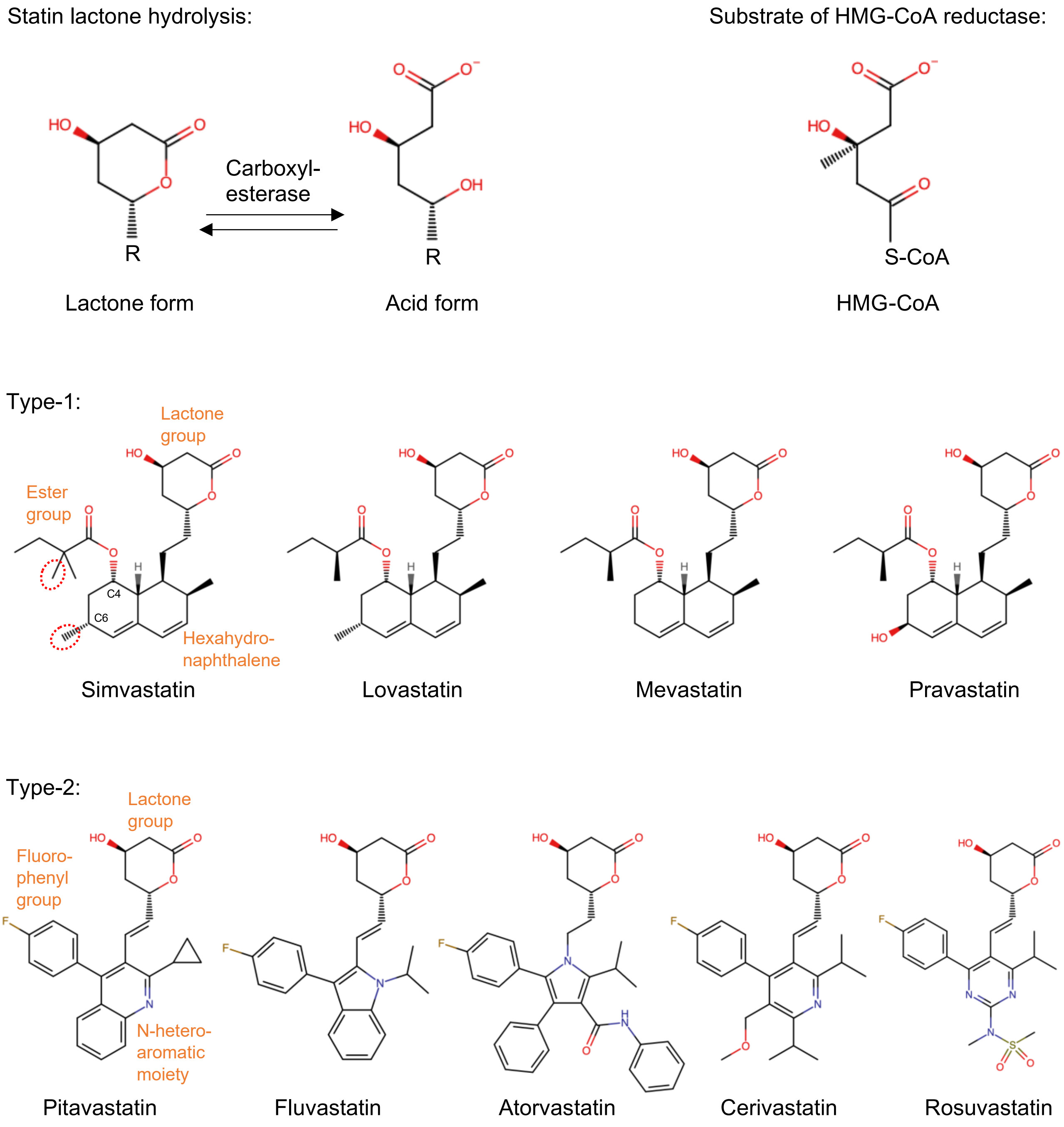

Supplemental Figure 1. Structural overview of the statin drug class. The lactone group is characteristic for statin drugs (top). The closed-ring lactone group of statins can be converted into the open-ring acid form (dihydroxyheptanoic acid) by hydrolysis. The hydrolysis of the statin lactone group is catalyzed by carboxylesterases. The dihydroxyheptanoic acid moiety resembles HMG-CoA, the substrate of the HMG-CoA reductase that is inhibited by statins. The statin drug class can be divided into type-1 (center) and type-2 statins (bottom). Besides the lactone group, type-1 statins typically possess a hydrophobic hexahydro-naphthalene core structure with a 2-methylbutyrate ester group at C4. An important member of the type-1 subgroup is simvastatin. In the simvastatin structure, the 2-methylbutyrate ester group is methylated to 2,2-dimethylbutyrate and hexahydro-naphthalene moiety is methylated at C6. Type-2 statins typically possess a N-heteroaromatic ring system as hydrophobic core structure

with a fluorophenyl group attached to it, which replaces the ester group of type-1 statins.

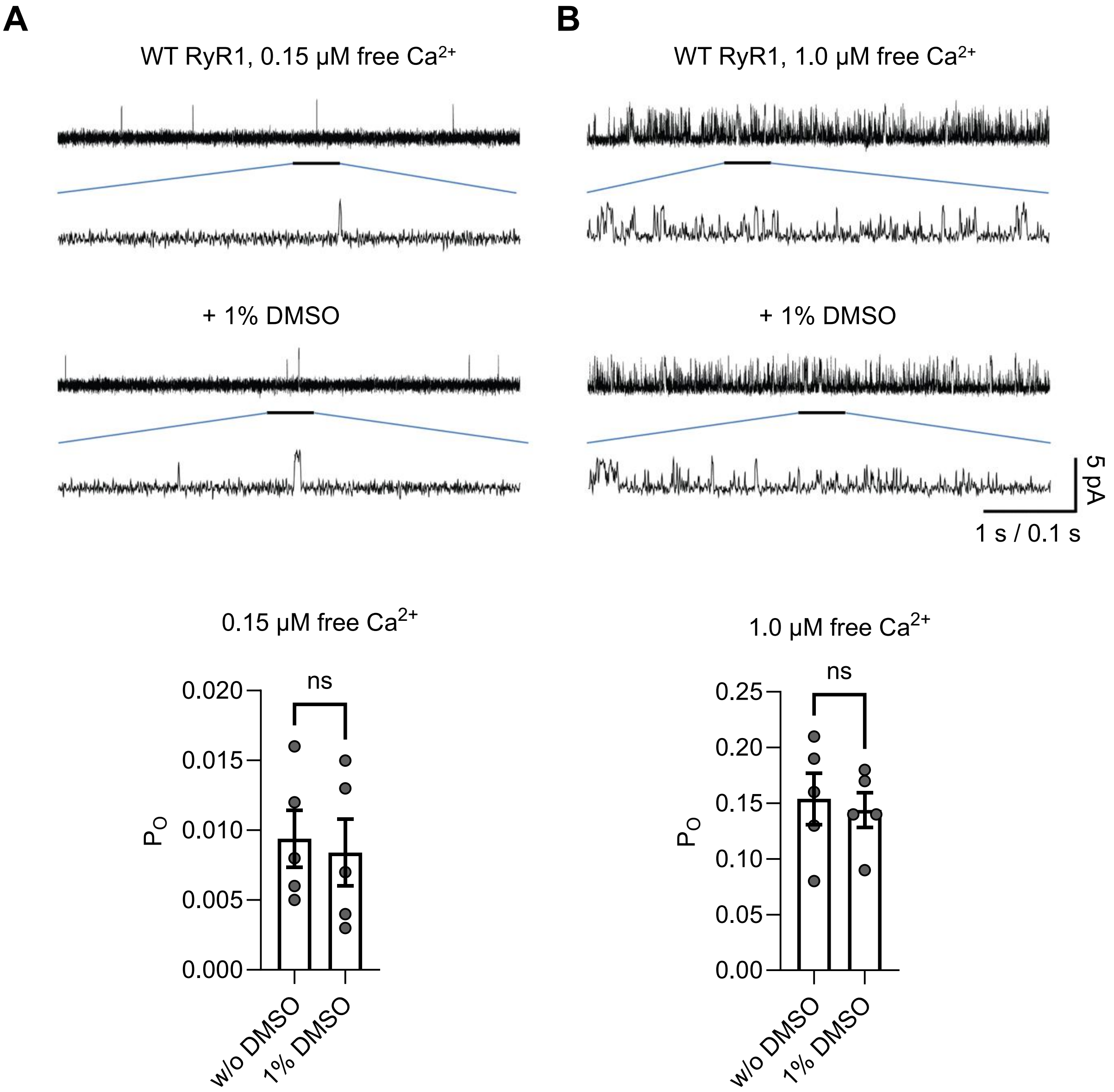

Supplemental Figure 2. RyR1 activity in presence and absence of 1% DMSO. Single channel recordings of skeletal muscle WT RyR1 channels reconstituted in planar lipid bilayers at (**A**) 0.15 µM or (**B**) 1.0 µM free Ca^{2+} in presence and absence of 1% DMSO. Single channel traces shown (top) are from the same RyR1 channel before (DMSO-free control condition) and shortly after addition of 1% DMSO. In the current traces, channel openings are represented as upward deflections from baseline (closed channel). Addition of 1% DMSO did not significantly alter the open probability (P_O) at 0.15 µM free Ca^{2+} (P_O = ~0.009 ± 0.002 without DMSO versus ~0.008 ± 0.002 with 1% DMSO; N = 5) or at 1.0 µM free Ca^{2+} (P_O = ~0.154 ± 0.023 without DMSO versus ~0.144 ± 0.016 with 1% DMSO; N = 5) (bottom). Data are expressed as mean ± SEM.

A

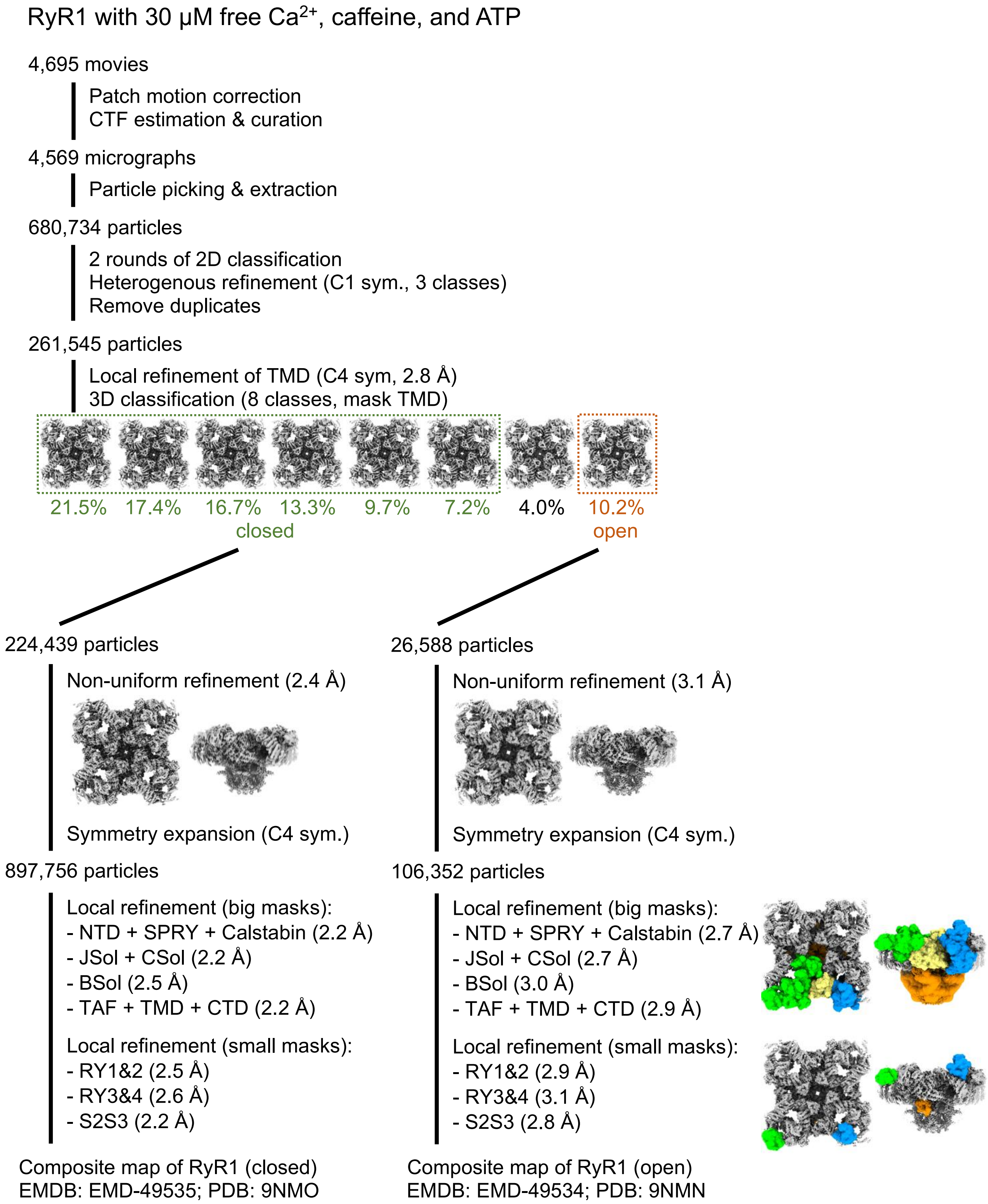
RyR1 with 30 µM free Ca2+, caffeine, and ATP
4,695 movies
Patch motion correction
CTF estimation & curation
4,569 micrographs
Particle picking & extraction
680,734 particles
2 rounds of 2D classification
Heterogenous refinement (C1 sym., 3 classes)
Remove duplicates
261,545 particles
Local refinement of TMD (C4 sym, 2.8 Å)
3D classification (8 classes, mask TMD)
21.5% 17.4% 16.7% 13.3% 9.7% 7.2% 4.0% 10.2%
closed
open
224,439 particles
Non-uniform refinement (2.4 Å)
Symmetry expansion (C4 sym.)
897,756 particles
Local refinement (big masks):
- NTD + SPRY + Calstabin (2.2 Å)
- JSol + CSol (2.2 Å)
- BSol (2.5 Å)
- TAF + TMD + CTD (2.2 Å)
Local refinement (small masks):
- RY1&2 (2.5 Å)
- RY3&4 (2.6 Å)
- S2S3 (2.2 Å)
Composite map of RyR1 (closed)
EMDB: EMD-49535; PDB: 9NMO
26,588 particles
Non-uniform refinement (3.1 Å)
Symmetry expansion (C4 sym.)
106,352 particles
Local refinement (big masks):
- NTD + SPRY + Calstabin (2.7 Å)
- JSol + CSol (2.7 Å)
- BSol (3.0 Å)
- TAF + TMD + CTD (2.9 Å)
Local refinement (small masks):
- RY1&2 (2.9 Å)
- RY3&4 (3.1 Å)
- S2S3 (2.8 Å)
Composite map of RyR1 (open)
EMDB: EMD-49534; PDB: 9NMN

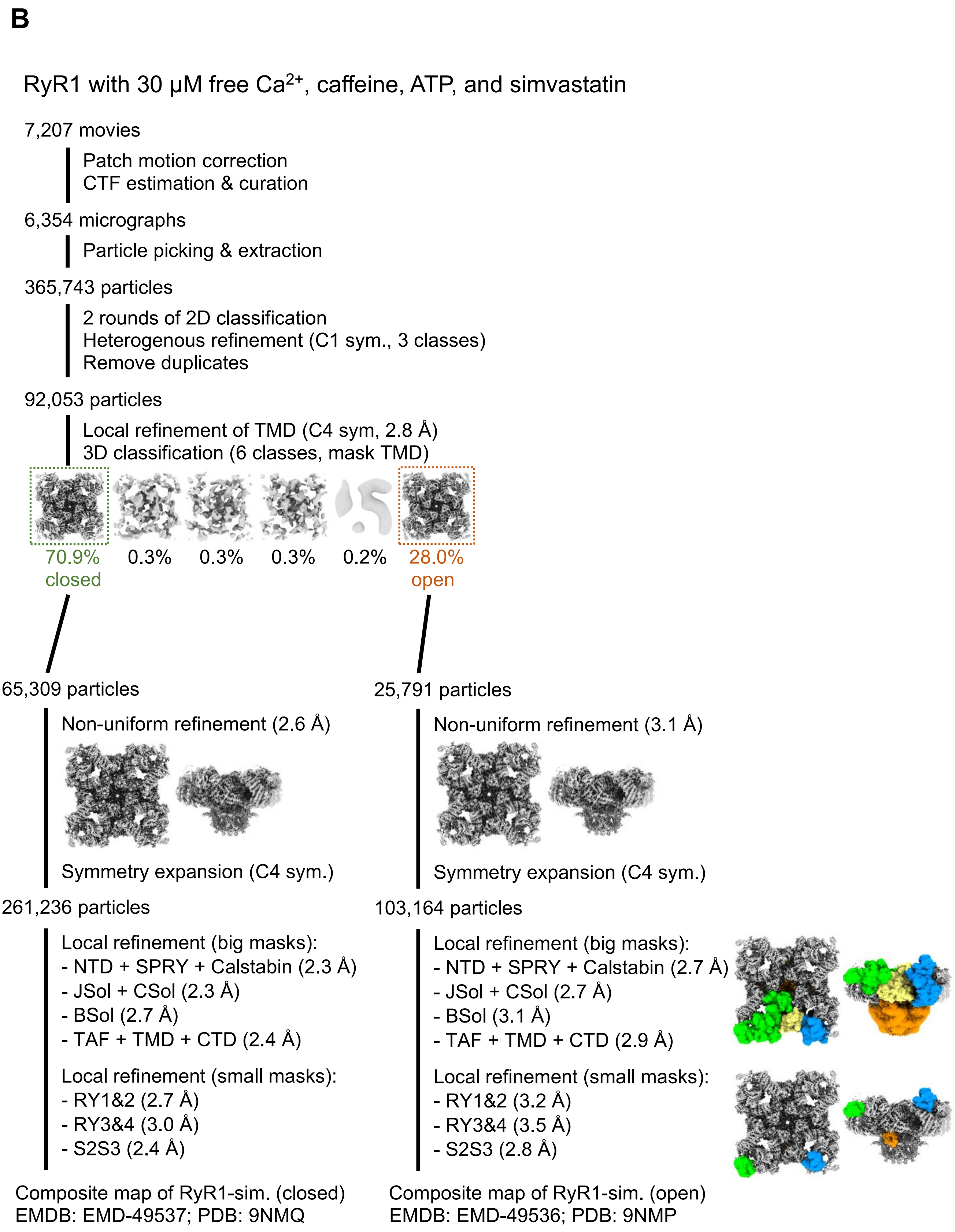
B
RyR1 with 30 µM free Ca2+, caffeine, ATP, and simvastatin
7,207 movies
Patch motion correction
CTF estimation & curation
6,354 micrographs
Particle picking & extraction
365,743 particles
2 rounds of 2D classification
Heterogenous refinement (C1 sym., 3 classes)
Remove duplicates
92,053 particles
Local refinement of TMD (C4 sym, 2.8 Å)
3D classification (6 classes, mask TMD)
70.9%
closed
0.3%
0.3%
0.3%
0.2%
28.0%
open
65,309 particles
Non-uniform refinement (2.6 Å)
Symmetry expansion (C4 sym.)
261,236 particles
Local refinement (big masks):
- NTD + SPRY + Calstabin (2.3 Å)
- JSol + CSol (2.3 Å)
- BSol (2.7 Å)
- TAF + TMD + CTD (2.4 Å)
Local refinement (small masks):
- RY1&2 (2.7 Å)
- RY3&4 (3.0 Å)
- S2S3 (2.4 Å)
Composite map of RyR1-sim. (closed)
EMDB: EMD-49537; PDB: 9NMQ
25,791 particles
Non-uniform refinement (3.1 Å)
Symmetry expansion (C4 sym.)
103,164 particles
Local refinement (big masks):
- NTD + SPRY + Calstabin (2.7 Å)
- JSol + CSol (2.7 Å)
- BSol (3.1 Å)
- TAF + TMD + CTD (2.9 Å)
Local refinement (small masks):
- RY1&2 (3.2 Å)
- RY3&4 (3.5 Å)
- S2S3 (2.8 Å)
Composite map of RyR1-sim. (open)
EMDB: EMD-49536; PDB: 9NMP

C

RyR1 with 5 mM EGTA

7,612 movies

| Patch motion correction
| CTF estimation & curation

6,636 micrographs

| Particle picking & extraction

333,640 particles

| 2 rounds of 2D classification
| Heterogenous refinement (C1 sym., 3 classes)
| Remove duplicates

129,073 particles

| Non-uniform refinement (2.9 Å)
| Symmetry expansion (C4 sym.)

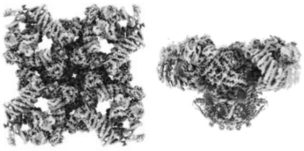

516,292 particles

| Local refinement (big masks):
| - NTD + SPRY + Calstabin (2.7 Å)
| - JSol + CSol (2.6 Å)
| - BSol (2.9 Å)
| - TAF + TMD + CTD (2.7 Å)

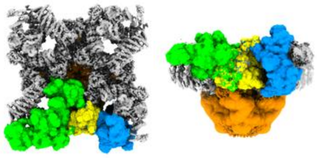

| Local refinement (small masks):
| - RY1&2 (2.9 Å)
| - RY3&4 (2.9 Å)
| - S2S3 (2.7 Å)

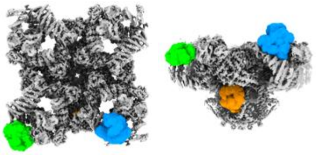

Composite map of RyR1 (apo)
EMDB: EMD-49538; PDB: 9NMR

Supplemental Figure 3. Cryo-EM data processing. Data processing workflows (performed in cryoSPARC) are shown for (**A**) the cryo-EM data set of RyR1 under activating conditions (30 µM free Ca^{2+}, caffeine, ATP) without simvastatin and (**B**) with simvastatin; (**C**) RyR1 (including the auxiliary transmembrane helix TMx) under non-activating conditions (5 mM EGTA).

A

RyR1 with 30 µM free Ca^{2+}, caffeine, and ATP; closed pore (PDB: 9NMO)

Non-uniform refinement:

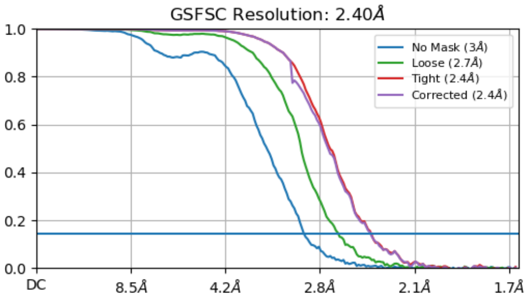

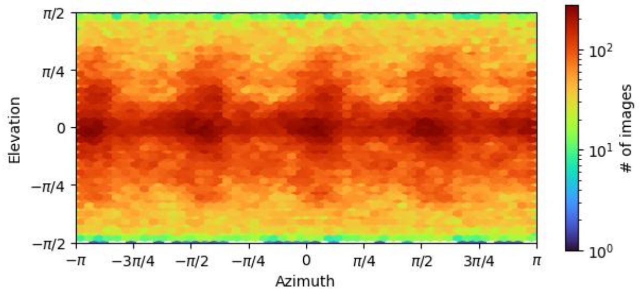

Local refinement of pore region (TAF + TMD + CTD) after C4-symmetry expansion:

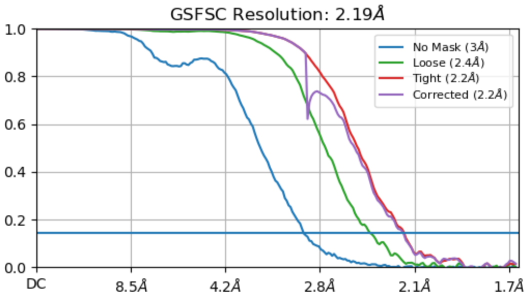

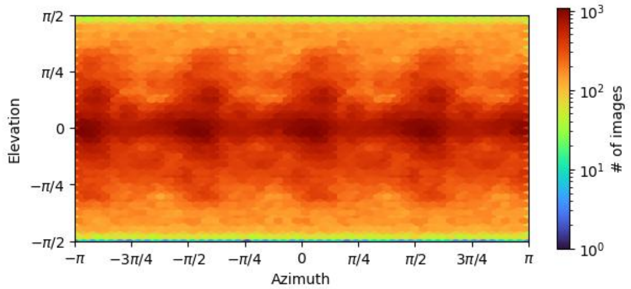

RyR1 with 30 µM free Ca^{2+}, caffeine, and ATP; open pore (PDB: 9NMN)

Non-uniform refinement:

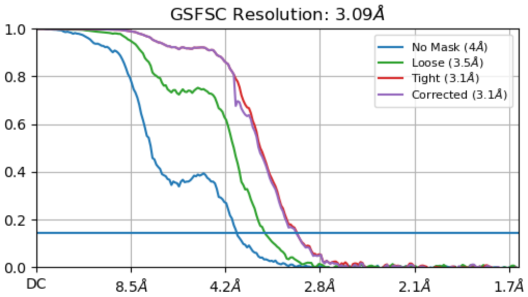

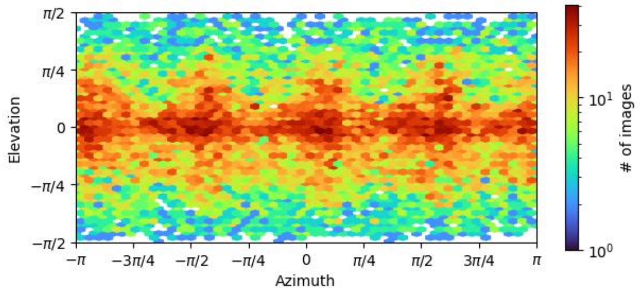

Local refinement of the pore region (TAF + TMD + CTD) after C4-symmetry expansion:

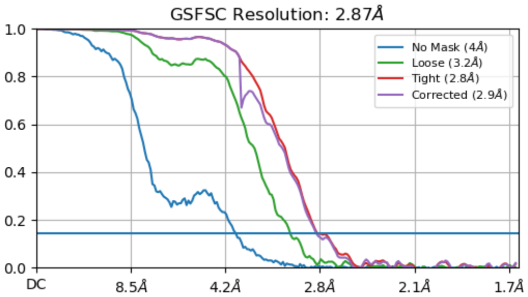

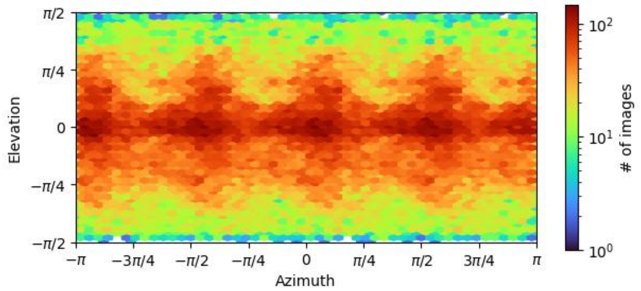

B

RyR1 with 30 µM free Ca^{2+}, caffeine, ATP, and simvastatin; closed pore (PDB: 9NMQ)

Non-uniform refinement:

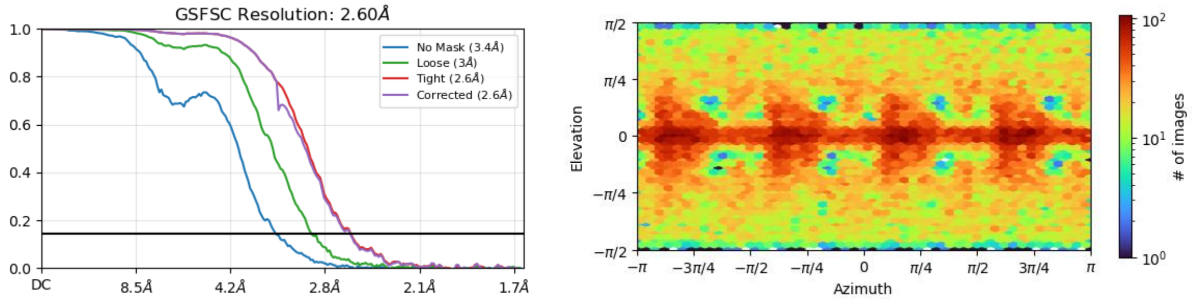

Local refinement of the pore region (TAF + TMD + CTD) after C4-symmetry expansion:

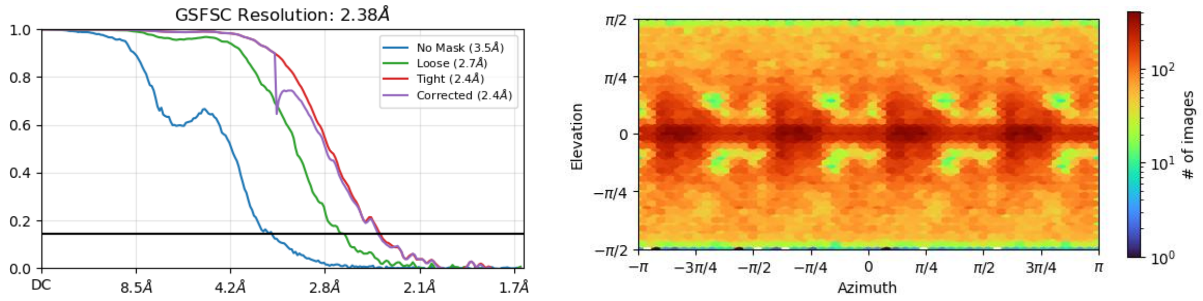

RyR1 with 30 µM free Ca^{2+}, caffeine, ATP, and simvastatin; open pore (PDB: 9NMP)

Non-uniform refinement:

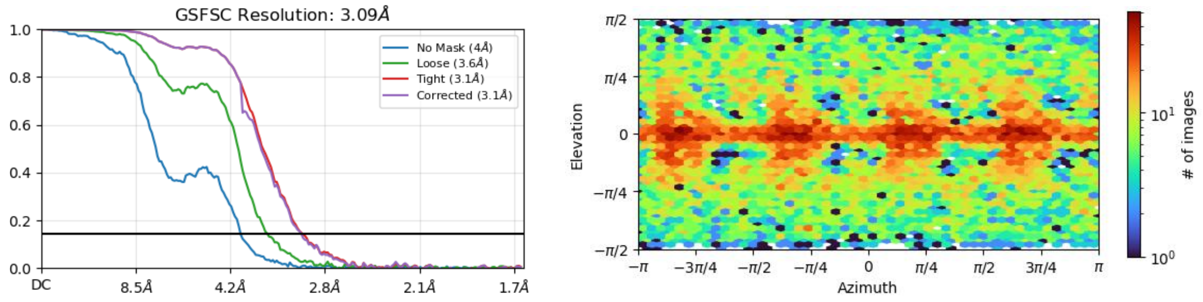

Local refinement of the pore region (TAF + TMD + CTD) after C4-symmetry expansion:

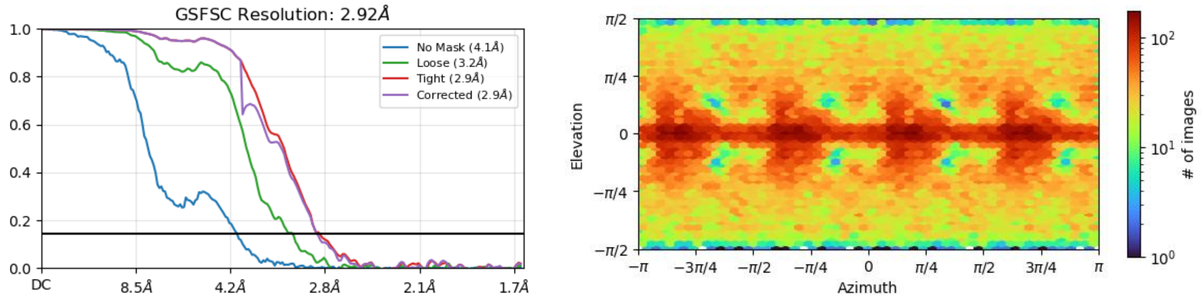

C

RyR1 with 5 mM EDTA; closed pore (PDB: 9NMR)

Non-uniform refinement:

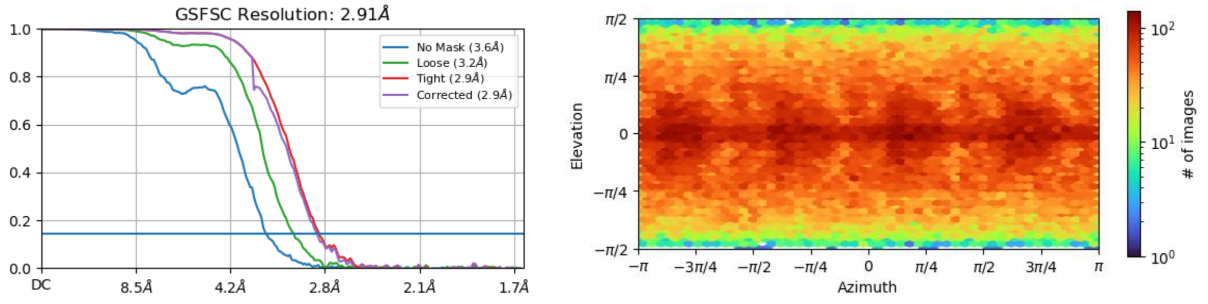

Local refinement of the pore region (TAF + TMD + CTD) after C4-symmetry expansion:

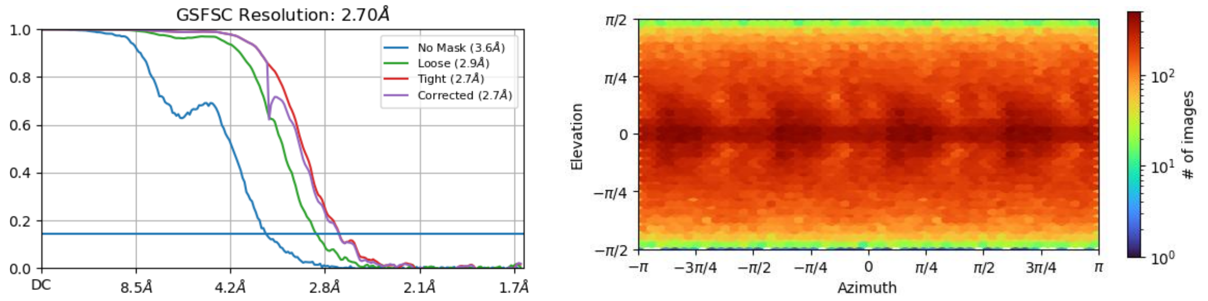

Supplemental Figure 4. FCS curves and orientation distribution heatmaps. Gold-standard FSC curves (left) and orientation distribution heatmaps (right) as determined during global non-uniform refinements and local refinements of the pore region (performed in cryoSPARC) are shown for (**A**) the cryo-EM data set of RyR1 under activating conditions (30 µM free Ca^{2+}, caffeine, ATP) without simvastatin and (**B**) with simvastatin; (**C**) RyR1 (including the auxiliary transmembrane helix TMx) under non-activating conditions (5 mM EGTA).

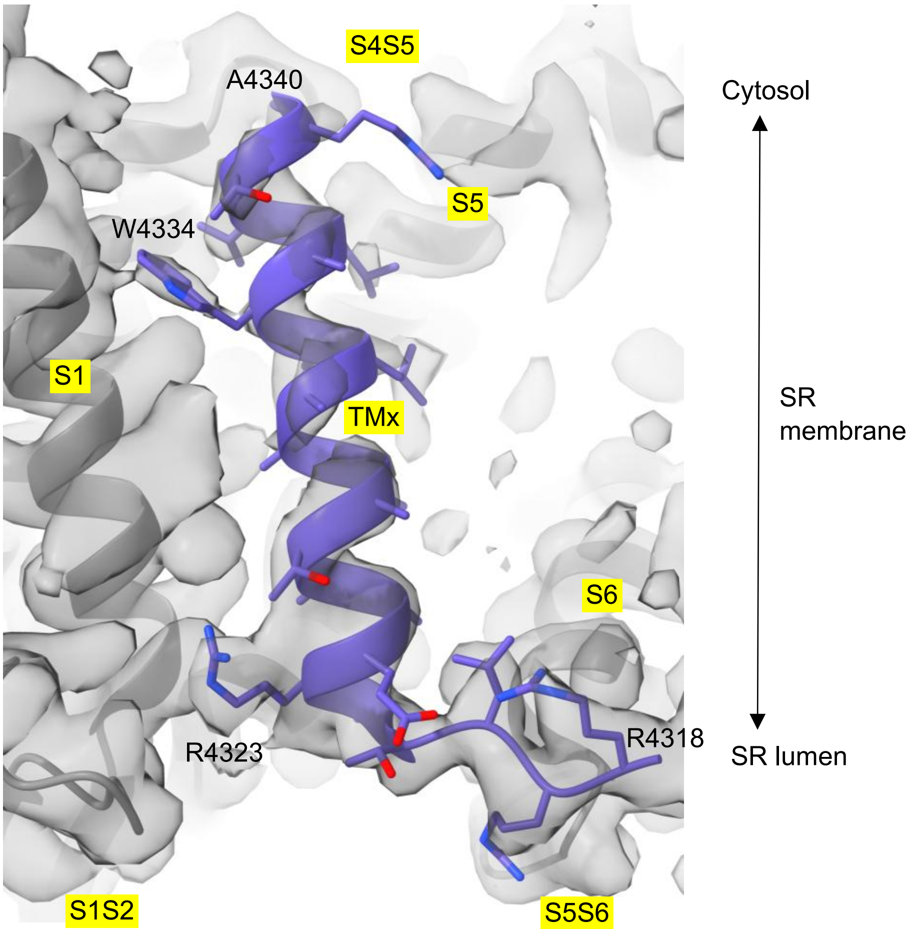

Supplemental Figure 5. Auxiliary transmembrane helix TMx of RyR1. TMx (colored; a.a. 4321-4340 in mouse RyR1; PDB: 9NMR) is located inside TMD between the pVSD and inner pore domain of adjacent RyR1 protomers. Next to the S5S6 loop, the N-terminal TMx region turns into an arginine-rich luminal extension (4311-RRRVRRLRRL-4320) that is only partially resolved. The C-terminus of TMx connects to an unresolved alanine/glycine-rich flexible loop (4340-AGGAGAGAAAG-4350) next to S4S5 at the cytosolic TMD site.

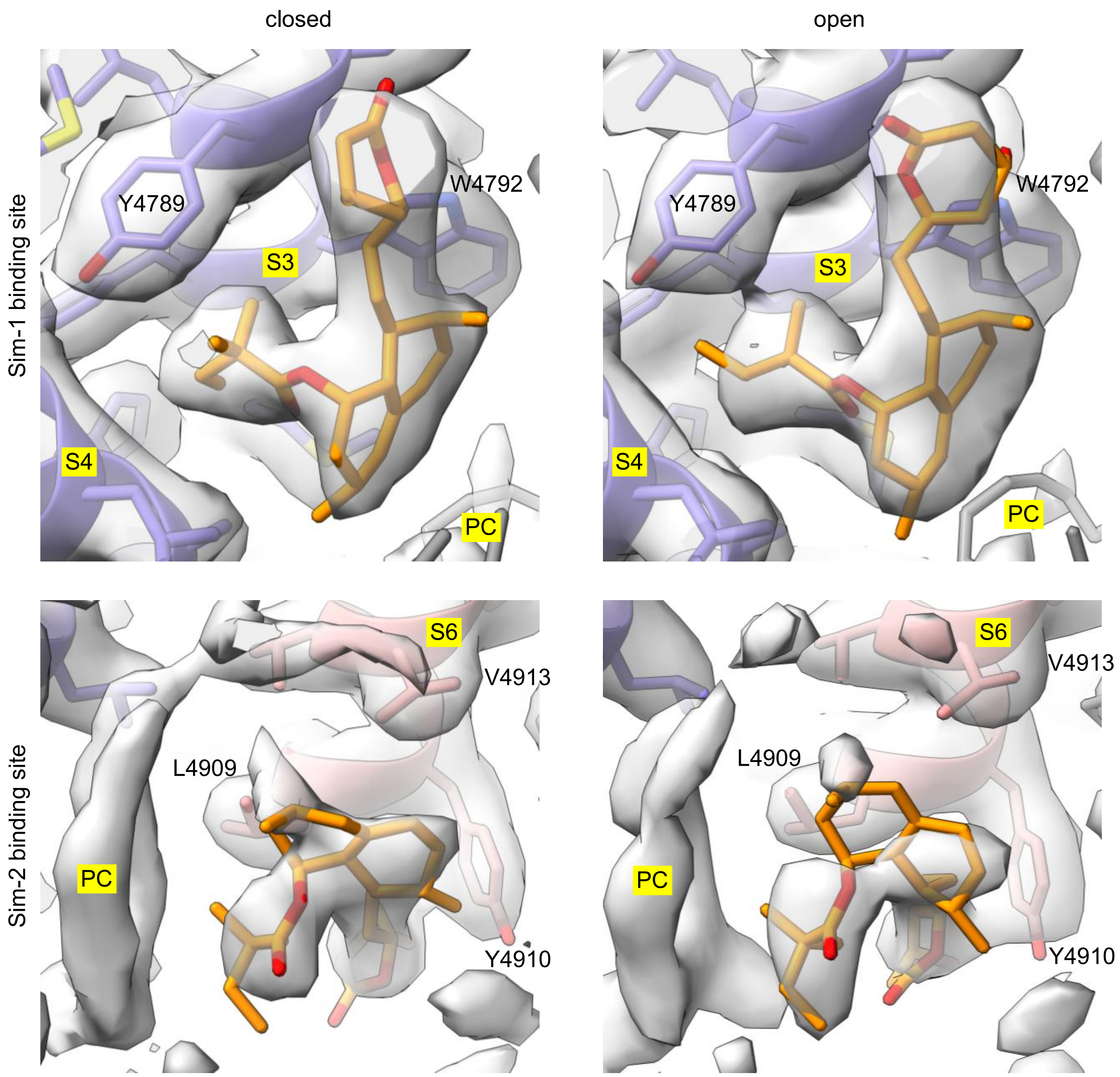

Supplemental Figure 6. Simvastatin binding sites Sim-1 and Sim-2 on RyR1 in closed and open conformational states. Simvastatin (orange) occupies Sim-1 (top) and Sim-2 (bottom) located in the pore region of RyR1 (grey map) in the closed (left) (PDB: 9NMQ) and open (right) (PDB: 9NMP) conformational states.

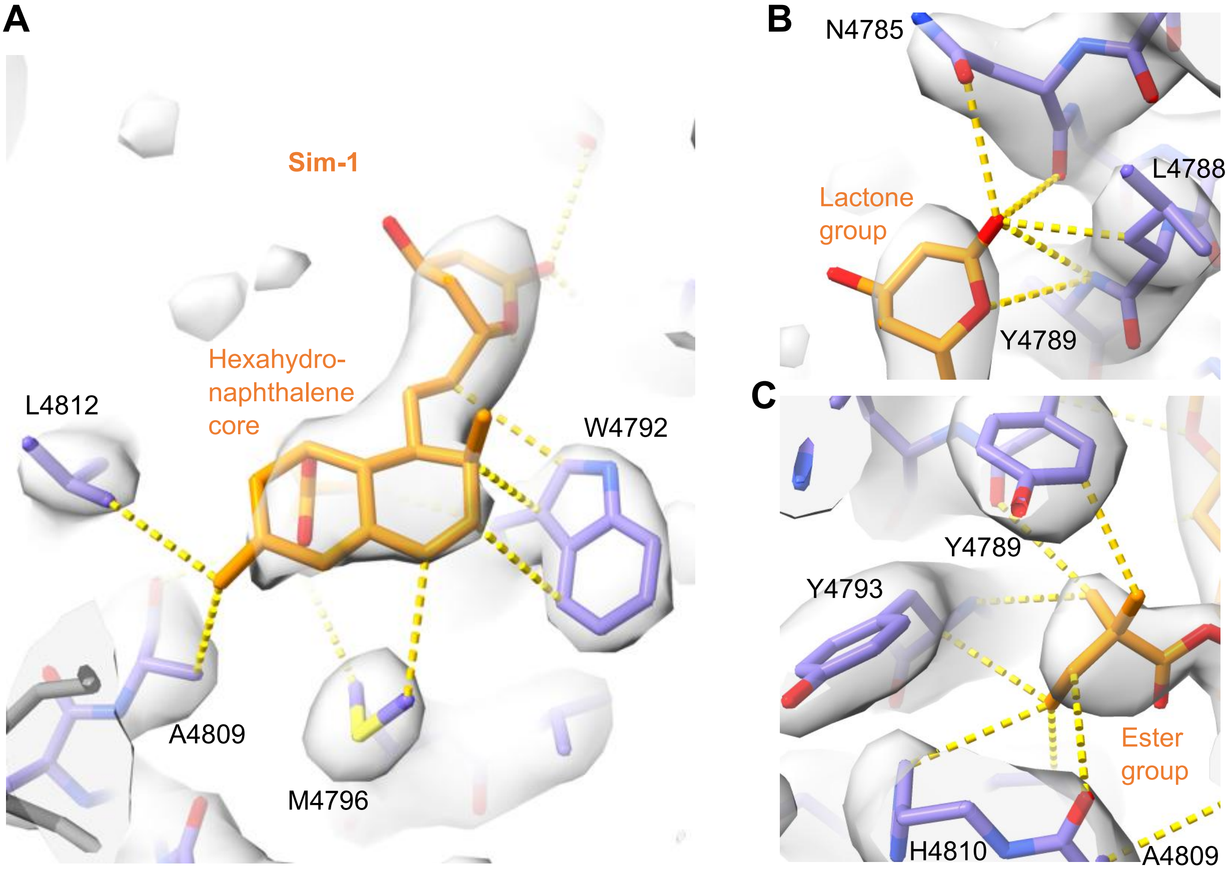

D

Mouse RyR1		Simvastatin		Distance
**Residue**	**Atom**	**Group**	**Atom**	**(Å)**
N4785	O	Lactone	O27	3.2
N4785	OD1	Lactone	O27	3.6
L4788	CB	Lactone	O27	3.3
Y4789	N	Lactone	O27	4.0
Y4789	N	Lactone	O28	4.0
Y4789	O	Ester	C5	3.8
Y4789	CE1	Ester	C4	3.2
W4792	CB	Ester	C5	3.8
W4792	CD1	Core	C21	3.7
W4792	CG	Core	C16	3.9
W4792	CE3	Core	C17	3.8
Y4793	CA	Ester	C1	3.9
Y4793	N	Ester	C5	3.9
M4796	CG	Ester	C1	3.4
M4796	CE	Core	C18	3.7
A4809	O	Ester	C2	3.7
A4809	CB	Core	C30	3.6
H4810	CD2	Ester	C1	4.0
L4812	CD1	Core	C30	3.9

Supplemental Figure 7. Contact sites between residues of RyR1 and functional groups of simvastatin in binding site Sim-1. (**A**-**C**) Local contacts of simvastatin (orange) with RyR1/Sim-1 (PDB: 9NMQ) are shown for (**A**) the hexahydro-naphthalene core, (**B**) the lactone group, and (**C**) the 2,2-dimethylbutyrate ester group of simvastatin. (**D**) List of RyR1 residues forming close contacts with simvastatin in the Sim-1 site (shown in **A**-**C**).

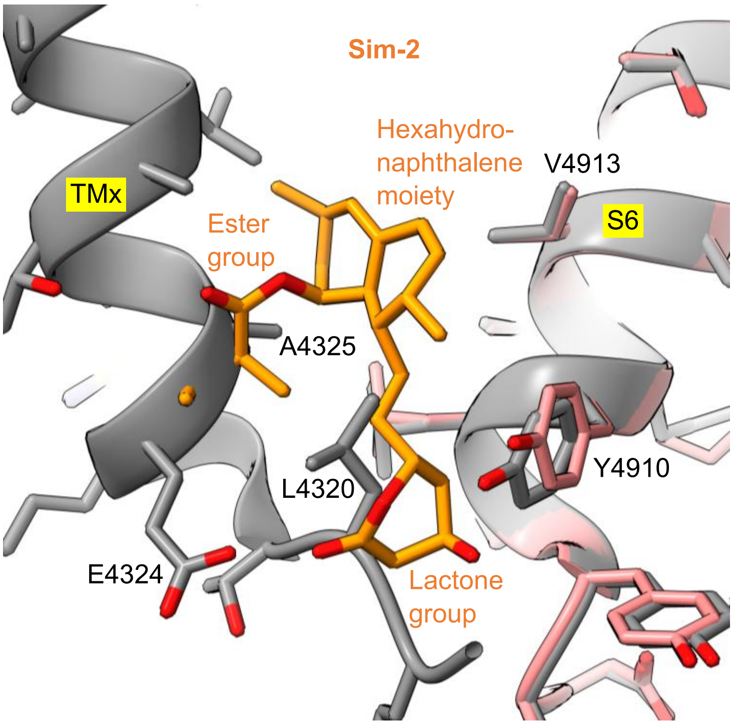

Supplemental Figure 8. Steric overlap between the auxiliary transmembrane helix TMx and simvastatin bound at the Sim-2 site. The superposition of RyR1 structures with simvastatin (colored; PDB: 9NMQ) and without simvastatin but including TMx (gray; PDB: 9NMR) reveals that the 2,2-dimethylbutyrate ester group of simvastatin (Sim-2) would sterically clash with the N-terminal TMx helix backbone at residues 4324-4325 and the lactone group with leucine L4320 near the luminal TMD surface. Hence, simvastatin binding at the Sim-2 site would displace TMx or require an induced fit in presence of TMx.

A

Sim-1:

```
                                S3                   S4
RyR1, mouse:   4778-FGVIFTDNSFLYLGWYMVMSLLGHYNNFFFAAHLLDIAMGV-4818
RyR1, human:   4781-FGVIFTDNSFLYLGWYMVMSLLGHYNNFFFAAHLLDIAMGV-4821
RyR1, rabbit:  4780-FGVIFTDNSFLYLGWYMVMSLLGHYNNFFFAAHLLDIAMGV-4820
RyR2, mouse:   4709-LGVVFTDNSFLYLAWYMTMSVLGHYNNFFFAAHLLDIAMGF-4749
RyR2, human:   4710-LGVVFTDNSFLYLAWYMTMSVLGHYNNFFFAAHLLDIAMGF-4750
RyR2, rabbit:  4712-LGVVFTDNSFLYLAWYMTMSILGHYNNFFFAAHLLDIAMGF-4752
RyR3, mouse:   4606-LGVVFTDNSFLYLAWYTTMSVLGHYNNFFFAAHLLDIAMGF-4646
RyR3, human:   4613-LGVVFTDNSFLYLAWYTTMSVLGHYNNFFFAAHLLDIAMGF-4653
RyR3, rabbit:  4615-LGVVFTDNSFLYLAWYTTMSVLGHYNNFFFAAHLLDIAMGF-4655
                    :**:*********.** .**:*******************.
```

Sim-2:

```
                                       S6
RyR1, mouse:   4898-EIEDPAGDEYELYRVVFDITFFFFVIVILLAIIQGLIIDAF-4938
RyR1, human:   4901-EIEDPAGDEYELYRVVFDITFFFFVIVILLAIIQGLIIDAF-4941
RyR1, rabbit:  4900-EIEDPAGDEYELYRVVFDITFFFFVIVILLAIIQGLIIDAF-4940
RyR2, mouse:   4829-EIEDPAGDEYEIYRIIFDITFFFFVIVILLAIIQGLIIDAF-4869
RyR2, human:   4830-EIEDPAGDEYEIYRIIFDITFFFFVIVILLAIIQGLIIDAF-4870
RyR2, rabbit:  4832-EIEDPAGDEYEIYRIIFDITFFFFVIVILLAIIQGLIIDAF-4872
RyR3, mouse:   4726-EIEDPAGDPYEMYRIVFDITFFFFVIVILLAIIQGLIIDAF-4766
RyR3, human:   4733-EIEDPAGDPYEMYRIVFDITFFFFVIVILLAIIQGLIIDAF-4773
RyR3, rabbit:  4735-EIEDPAGDPYEMYRIVFDITFFFFVIVILLAIIQGLIIDAF-4775
                    ******** **:**::*************************
```

B

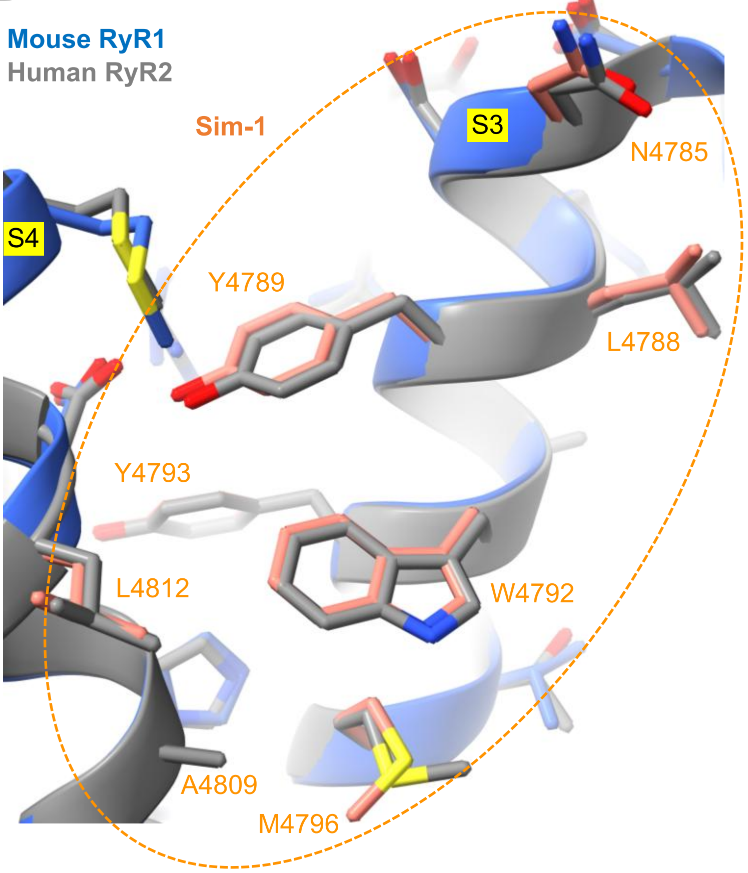

C

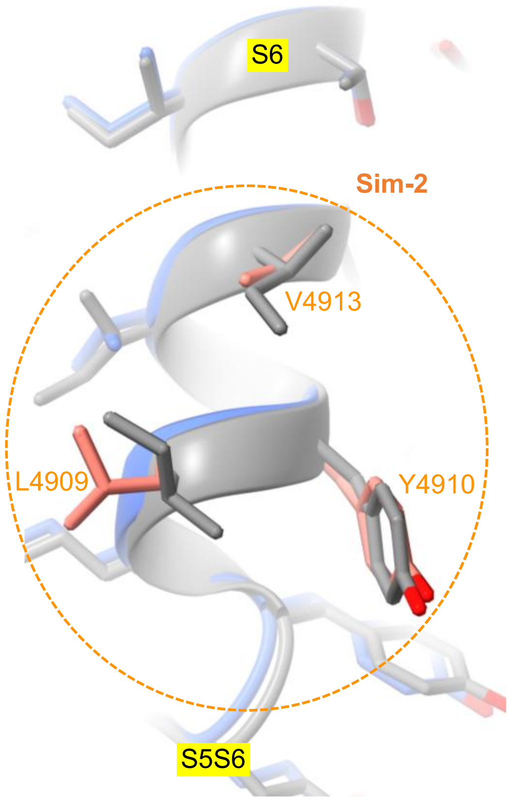

Supplemental Figure 9. Evolutionary conservation of the simvastatin binding sites across mammalian species and isoforms of RyR. (**A**) Multiple sequence alignments between the RyR1, RyR2, and RyR3 isoforms from mouse, human, and rabbit at the simvastatin binding sites Sim-1 and Sim-2. Residues forming Sim-1 (Figure 1B) or Sim-2 (Figure 1C) are located in highly conserved sequence regions of

the TM helices S3 and S4 or N-terminal S6, respectively (identical residues in red; conserved residues in orange). The alignments show complete sequence identity for Sim-1 and high conservation for Sim-2. Protein sequences from UniProt database: mouse RyR1, ID: E9PZQ0; human RyR1, ID: P21817-1; rabbit RyR1, ID: P11716; mouse RyR2, ID: E9Q401; human RyR2, ID: Q92736-1; rabbit RyR2, ID: P30957; mouse RyR3, ID: A2AGL3-1; human RyR3, ID: Q15413-1; rabbit RyR3, ID: Q9TS33-1. Asterisks, colons, and periods indicate identical residues, highly conserved residues, and semi-conserved residues, respectively. (**B**-**C**) Structural comparison of the unoccupied Sim-1 (**B**) and Sim-2 (**C**) sites in mouse RyR1 (blue) (PDB: 9MNO) with human RyR2 (gray) (PDB: 7UA5). Residues forming the Sim-1 and Sim-2 sites (red) in mouse RyR1 are structurally conserved in human RyR2.

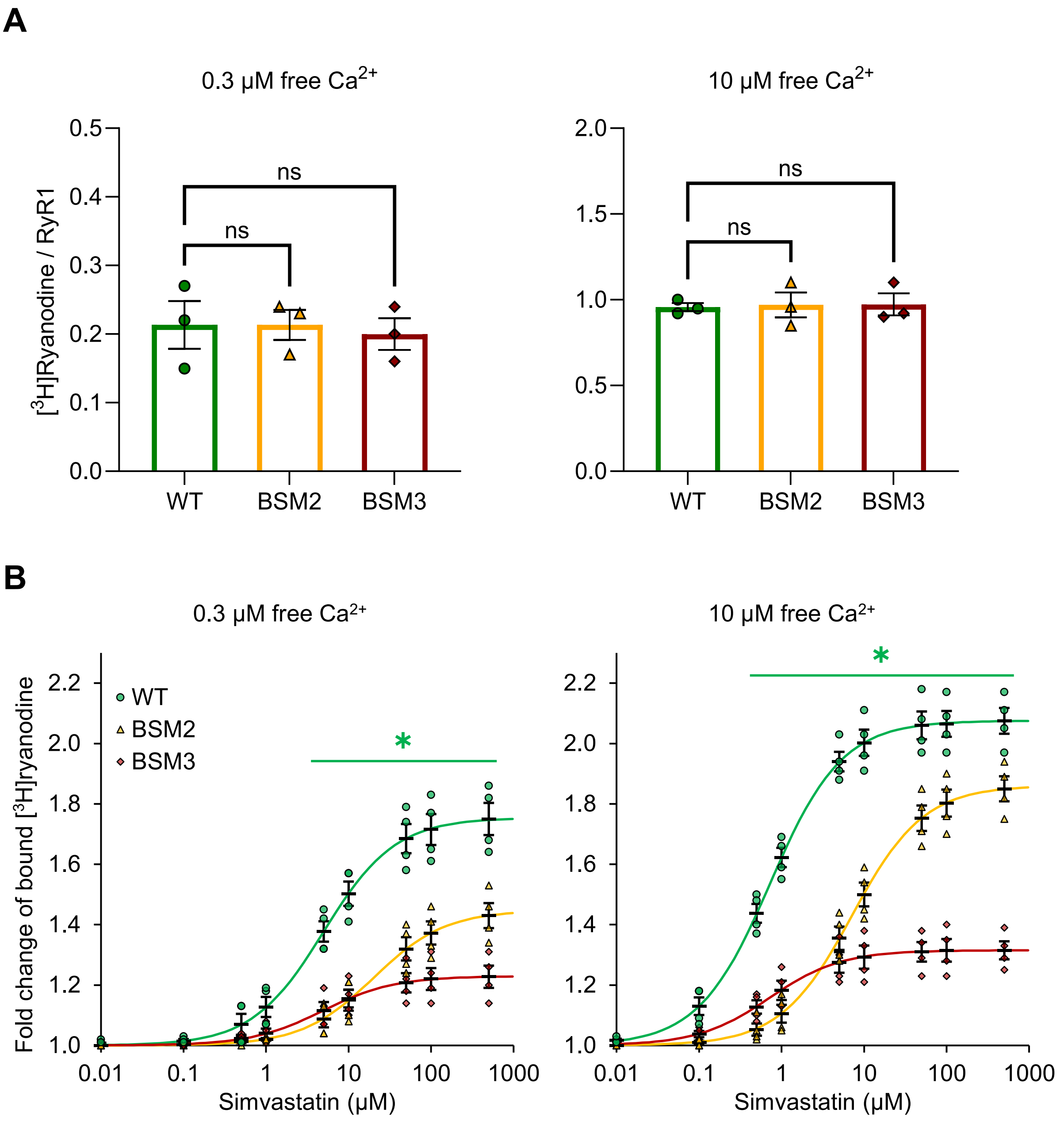

	0.3 µM free Ca^{2+}			10 µM free Ca^{2+}		
	EC_{50} (µM)	E_{max}	R^2	EC_{50} (µM)	E_{max}	R^2
WT	4.99 ± 0.89	1.75 ± 0.02	0.953	0.73 ± 0.09	2.08 ± 0.02	0.976
BSM2	20.03 ± 4.90	1.45 ± 0.02	0.923	7.15 ± 0.96	1.86 ± 0.02	0.972
BSM3	4.85 ± 1.87	1.23 ± 0.02	0.812	0.72 ± 0.23	1.32 ± 0.01	0.844

Supplemental Figure 10. Simvastatin dose response of Sim-1/Sim-2 binding site mutants in [^{3}H]ryanodine binding assays. (**A**) [^{3}H]Ryanodine binding data are shown for recombinant WT RyR1 (green) and Sim-1/Sim-2 binding site mutants (BSM2: Y4789A+W4792A in yellow; BSM3: Y4910A in red). These mutations did not cause a significant difference in [^{3}H]ryanodine binding at 0.3 µM (left) or 10 µM free Ca^{2+} (right), implying similar activities of BSM2 and BSM3 compared to WT RyR1 in absence of simvastatin. (**B**) Dose response to simvastatin acid at concentrations

ranging from 0.01 to 500 µM at 0.3 µM (top left) or 10 µM free Ca^{2+} (top right). E_{max} and EC_{50} values were determined from the simvastatin dose response curves of WT RyR1, BSM2, and BSM3 (bottom). Both BSM2 and BSM3 significantly reduced the simvastatin dose response. Data are expressed as mean ± SEM. 2-way ANOVA, *$P < 0.05$.

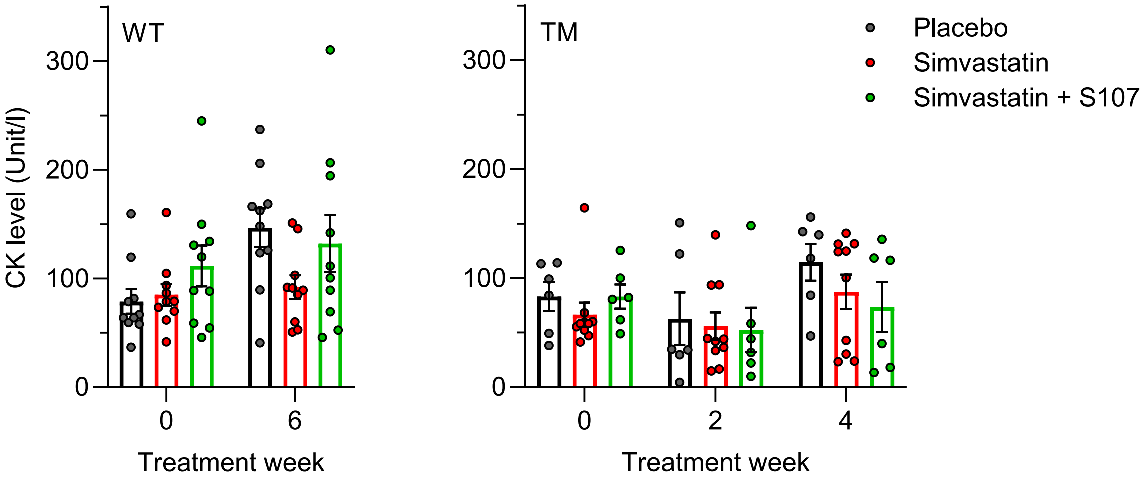

Supplemental Figure 11. CK blood levels in WT versus heterozygous TM mice treated with simvastatin. WT or heterozygous TM mice were treated with placebo (black), ~50 mg/kg/day simvastatin (red), or the latter together with ~50 mg/kg/day S107 (green). Blood samples were taken before (week 0), during (week 2 for TM) and after treatment (week 6 for WT, week 4 for TM). CK blood levels expressed as mean ± SEM are plotted as bar graphs. The CK blood levels in WT (left) or heterozygous TM mice (right) were not significantly altered by the simvastatin treatment.

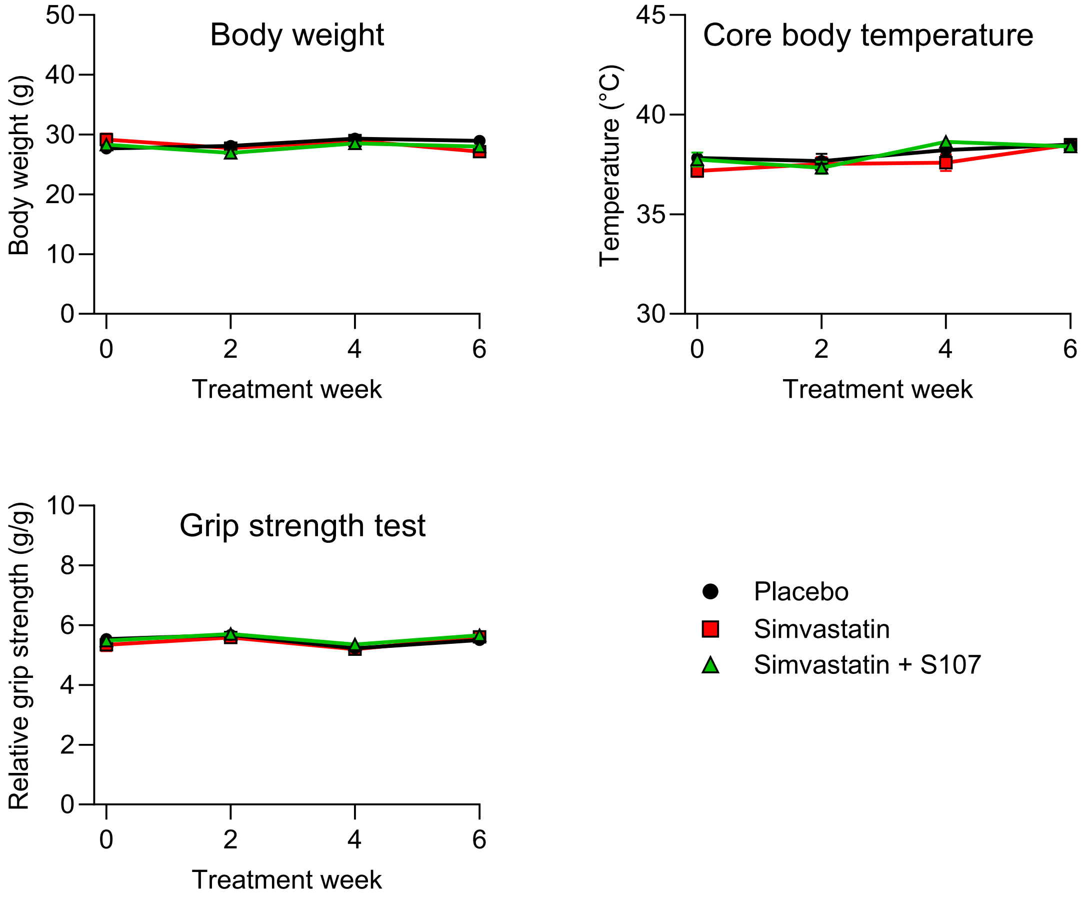

Supplemental Figure 12. *In vivo* behavioral data of WT mice treated with simvastatin. WT mice were treated with placebo (N = 10), 50 mg/kg/day simvastatin (N =10), or simvastatin + S107 (both ~50 mg/kg/day; N = 10) for 6 weeks. The body weight (top left), core body temperature (top right), and grip strength (bottom) of WT mice did not significantly alter upon simvastatin treatment. Data are expressed as mean ± SEM.

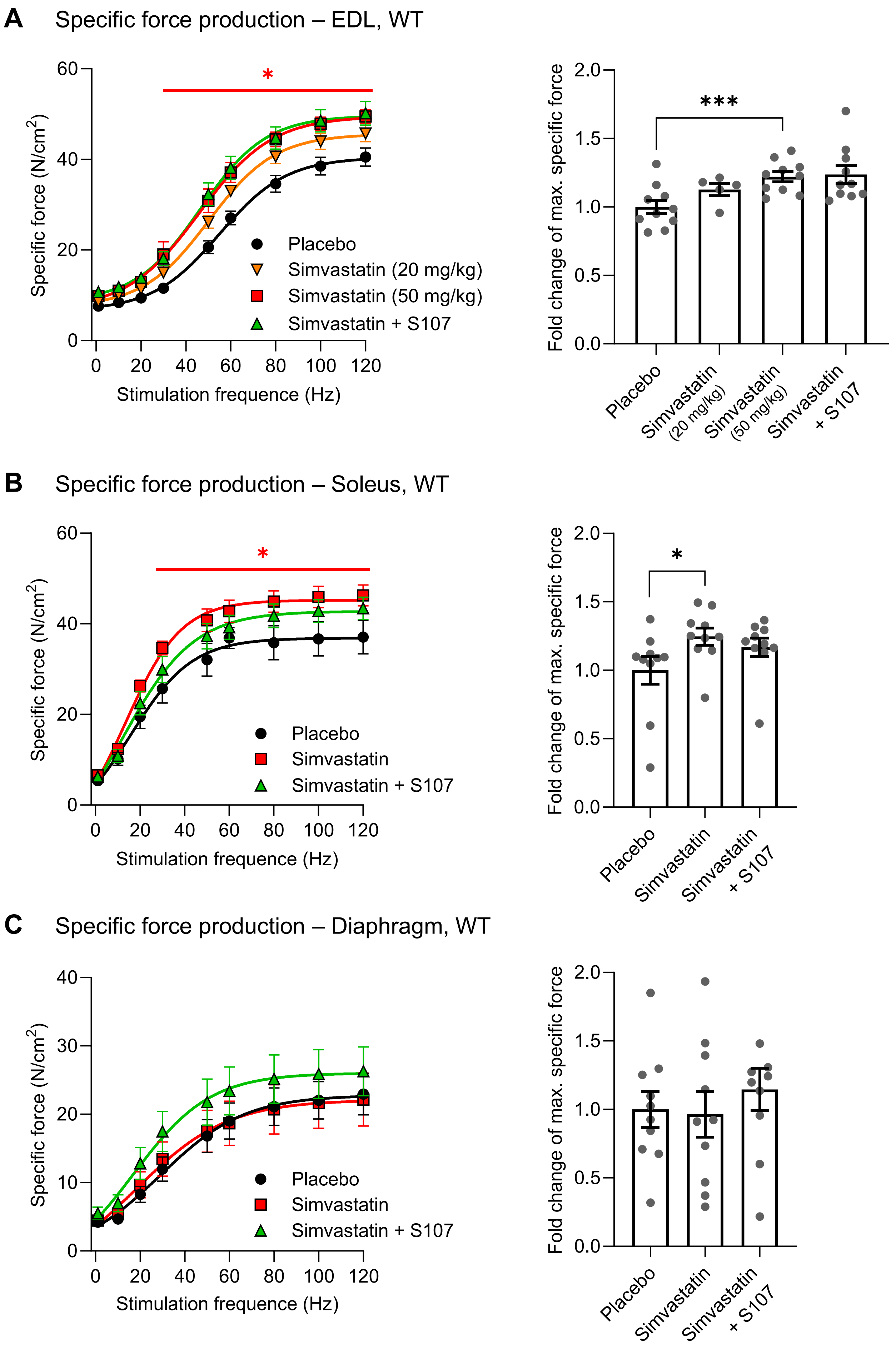
A
Specific force production – EDL, WT
Specific force (N/cm^2)
Stimulation frequence (Hz)
*
Placebo
Simvastatin (20 mg/kg)
Simvastatin (50 mg/kg)
Simvastatin + S107
Fold change of max. specific force
***
Placebo
Simvastatin (20 mg/kg)
Simvastatin (50 mg/kg)
Simvastatin + S107
B
Specific force production – Soleus, WT
Specific force (N/cm^2)
Stimulation frequence (Hz)
*
Placebo
Simvastatin
Simvastatin + S107
Fold change of max. specific force
*
Placebo
Simvastatin
Simvastatin + S107
C
Specific force production – Diaphragm, WT
Specific force (N/cm^2)
Stimulation frequence (Hz)
Placebo
Simvastatin
Simvastatin + S107
Fold change of max. specific force
Placebo
Simvastatin
Simvastatin + S107

Supplemental Figure 13. Specific force assessment of *ex vivo* muscles from WT mice treated with simvastatin. WT mice were treated with placebo (N = 10), ~20 or ~50 mg/kg/day simvastatin (N = 10), or simvastatin + S107 (both ~50 mg/kg/day; N = 10). (**A**-**C**) Specific force production plotted against the electrical stimulation frequence (left) and the corresponding fold changes of the maximal specific force (right) are shown *ex vivo* for (**A**) EDL, (**B**) soleus and (**C**) diaphragm. Simvastatin treatment significantly improved the specific force production of EDL and soleus at stimulation frequences ≥ 30 Hz compared to placebo. Data are expressed as mean ± SEM. 2-way ANOVA, *P < 0.05, ***P < 0.001.

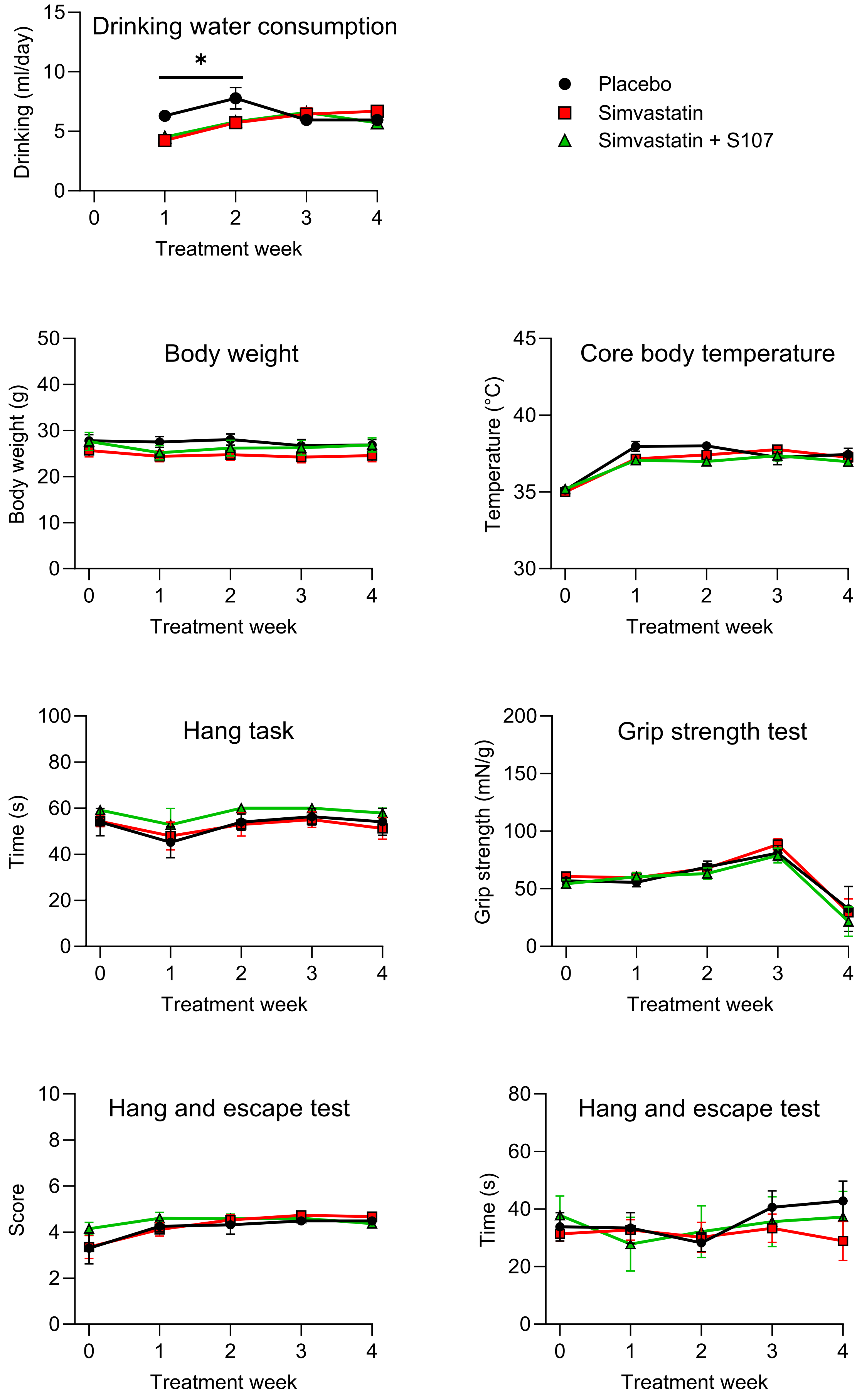
Drinking water consumption
Drinking (ml/day)
*
Treatment week
Placebo
Simvastatin
Simvastatin + S107
Body weight
Body weight (g)
Treatment week
Core body temperature
Temperature (°C)
Treatment week
Hang task
Time (s)
Treatment week
Grip strength test
Grip strength (mN/g)
Treatment week
Hang and escape test
Score
Treatment week
Hang and escape test
Time (s)
Treatment week

Supplemental Figure 14. *In vivo* behavioral data of heterozygous TM mice treated with simvastatin. Heterozygous TM mice were treated with placebo (N = 6), 50 mg/kg/day simvastatin (N =10), or simvastatin + S107 (both ~50 mg/kg/day; N = 6) for 4 weeks. Shown are the consumption of drinking water (top left), body weight (2nd row left), core body temperature (2nd row right), hang task time (3rd row left), grip strength (3rd row right), hang and escape score (bottom left) and time (bottom right). Data are expressed as mean ± SEM. The core body temperature was measured using an intraperitoneally implanted UID temperature microchip. The initial temperature values (week 0) were lower due to anesthesia. No significant differences were detected between placebo and simvastatin-treated groups in these *in vivo* behavioral data sets except for the early water consumption. Drinking water without simvastatin (placebo) was significantly preferred during the first two treatment weeks (2-way ANOVA, *P < 0.05). In the hang and escape test, the simvastatin-treated group showed a trend to decreasing hang and escape times in the final treatment week 4 compared to placebo.

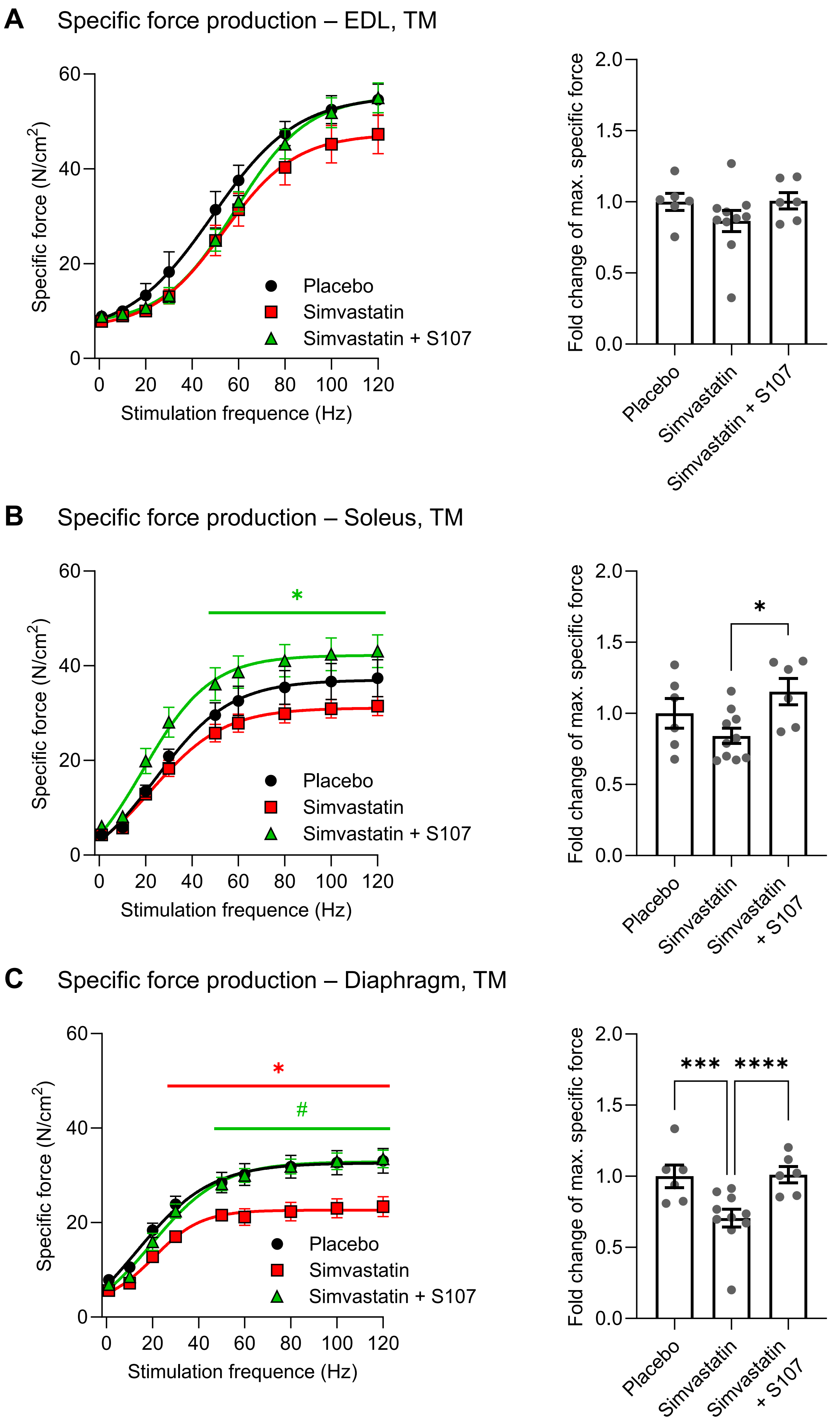
A
Specific force production – EDL, TM
Specific force (N/cm2)
Stimulation frequence (Hz)
Placebo
Simvastatin
Simvastatin + S107
Fold change of max. specific force
Placebo
Simvastatin
Simvastatin + S107
B
Specific force production – Soleus, TM
Specific force (N/cm2)
Stimulation frequence (Hz)
*
Placebo
Simvastatin
Simvastatin + S107
Fold change of max. specific force
*
Placebo
Simvastatin
Simvastatin + S107
C
Specific force production – Diaphragm, TM
Specific force (N/cm2)
Stimulation frequence (Hz)
*
#
Placebo
Simvastatin
Simvastatin + S107
Fold change of max. specific force
***
****
Placebo
Simvastatin
Simvastatin + S107

Supplemental Figure 15. Specific force assessment of *ex vivo* muscles from heterozygous TM mice treated with simvastatin. Heterozygous TM mice were treated with placebo (N = 6), 50 mg/kg/day simvastatin (N =10), or simvastatin + S107 (both ~50 mg/kg/day; N = 6) for 4 weeks. (**A**-**C**) The specific force production plotted against the electrical stimulation frequence (left) and the corresponding fold changes of the maximal specific force (right) are shown *ex vivo* for (**A**) EDL, (**B**) soleus and (**C**) diaphragm. Simvastatin treatment resulted in significantly weaker diaphragmatic forces at stimulation frequences ≥ 30 Hz, while EDL and soleus showed a similar decreasing trend following simvastatin treatment at maximal specific forces. Addition of S107 significantly increased the specific force production of the soleus and diaphragm in presence of simvastatin compared to simvastatin alone at stimulation frequences ≥ 50 Hz. Data are expressed as mean ± SEM. 2-way ANOVA, *P < 0.05, ***P < 0.001, ****P < 0.0001.

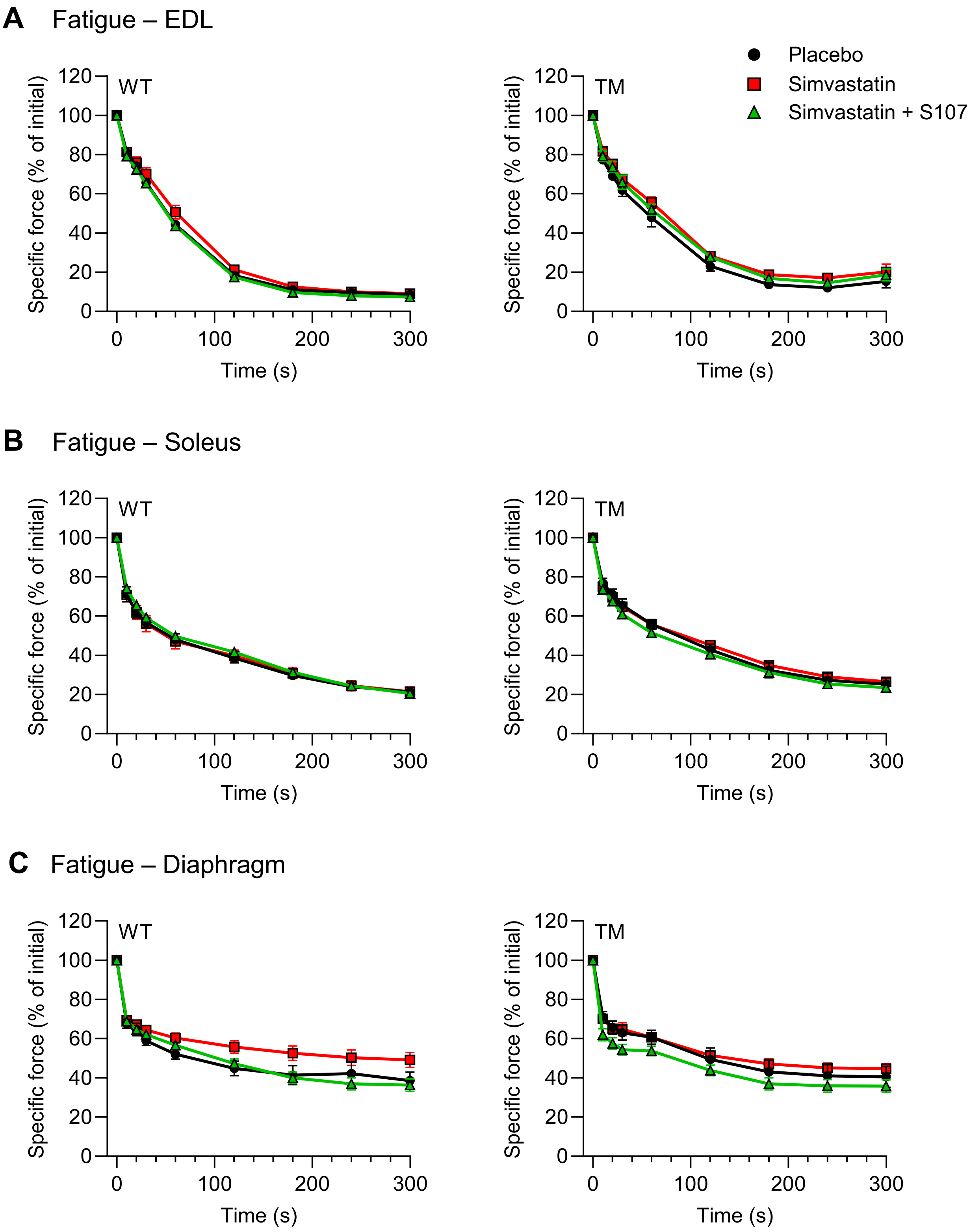

Supplemental Figure 16. Fatigue assessment of *ex vivo* muscles from WT and heterozygous TM mice treated with simvastatin. WT and heterozygous TM mice were treated with placebo (N = 6 WT / 10 TM), 50 mg/kg/day simvastatin (N = 10 WT / 10 TM), or simvastatin + S107 (both ~50 mg/kg/day; N = 6 WT / 10 TM) for 6 weeks (WT) or 4 weeks (TM). (**A**-**C**) The percentage of the initial specific force is plotted

against the time of the repetitive electrical stimulation protocol (at 30 Hz) showing fatigue curves of the *ex vivo* (**A**) EDL, (**B**) soleus and (**C**) diaphragm. Data are expressed as mean ± SEM. The fatigue curves were not significantly affected by the simvastatin treatment of WT (left) or heterozygous TM mice (right).

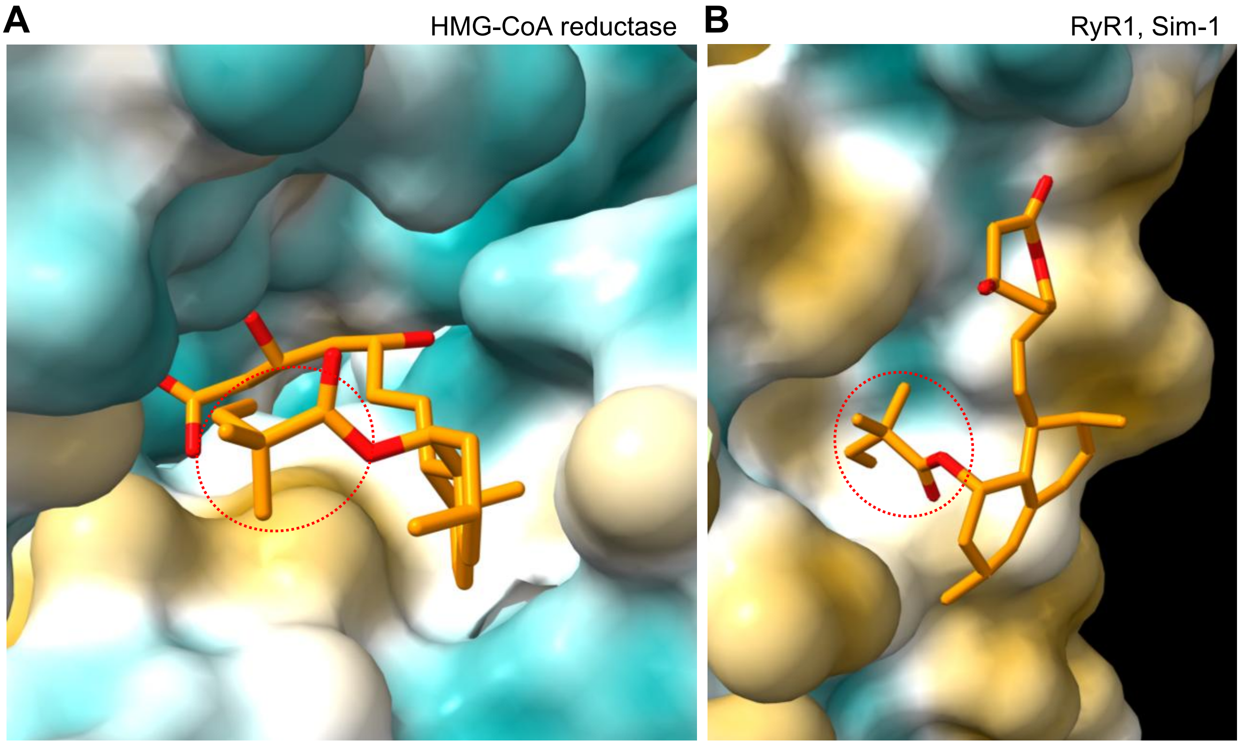

Supplemental Figure 17. Structural comparison of simvastatin binding sites on HMG-CoA reductase and RyR1. Binding modes of simvastatin (orange) are shown on HMG-CoA reductase in the HMG-CoA binding pocket (PDB: 1HW9) and on RyR1 in Sim-1 (PDB: 9NMQ). Surface coloring of the binding sites indicates hydrophilic and hydrophobic potentials in cyan and goldenrod, respectively. (**A**) The simvastatin binding pose on the HMG-CoA reductase reveals tight interactions mediated by the hydrophobic hexahydro-naphthalene core and the hydrophilic dihydroxyheptanoic acid moiety (opened lactone group) protruding into the substrate binding pocket, while the pocket is more spacious behind the 2,2-dimethylbutyrate ester group (red circle). (**B**) On RyR1, the 2,2-dimethylbutyrate ester group of simvastatin occupies the hydrophobic pocket of Sim-1, while the lactone group is less tightly engaged.

A

Dataset	RyR1, closed (EGTA only)	RyR1, closed	RyR1, open	RyR1, closed + simvastatin	RyR1, open + simvastatin
PDB ID	9NMR	9NMO	9NMN	9NMQ	9NMP
**Data collection**					
Microscope	FEI Titan Krios	FEI Titan Krios	FEI Titan Krios	FEI Titan Krios	FEI Titan Krios
Detector	Gatan K3	Gatan K3	Gatan K3	Gatan K3	Gatan K3
Voltage	300 kV	300 kV	300 kV	300 kV	300 kV
Magnification	105,000 x	105,000 x	105,000 x	105,000 x	105,000 x
Exposure	58 $e^{-}/Å^{2}$	58 $e^{-}/Å^{2}$	58 $e^{-}/Å^{2}$	58 $e^{-}/Å^{2}$	58 $e^{-}/Å^{2}$
Defocus range	-0.4 to -1.2 μM	-0.4 to -1.2 μM	-0.4 to -1.2 μM	-0.4 to -1.2 μM	-0.4 to -1.2 μM
Pixel size	~0.83 Å	~0.83 Å	~0.83 Å	~0.83 Å	~0.83 Å
**Processing**					
Software	cryoSPARK	cryoSPARK	cryoSPARK	cryoSPARK	cryoSPARK
Symmetry	C4	C4	C4	C4	C4
Particles (N)	129,073	224,439	26,588	65,309	25,791
Resolution	2.9 Å	2.4 Å	3.1 Å	2.6 Å	3.1 Å
**Model building**					
Software	Coot	Coot	Coot	Coot	Coot
**Model composition**					
Peptide chains	4+4	4+4	4+4	4+4	4+4
Residues	18016	17920	17920	17920	17920
**Model refinement**					
Software	Phenix	Phenix	Phenix	Phenix	Phenix
**Validation**					
MolProbity score	1.52	1.55	1.54	1.99	1.79
Clashscore	6.07	4.57	603	7.25	9.16
Rotamer outliers	0.56%	1.88%	0.18%	3.64%	1.64%
**RMSD**					
Bond lengths	0.005 Å	0.004 Å	0.005 Å	0.006 Å	0.006 Å
Bond angles	0.695°	0.689°	0.671°	0.771°	0.794°
**Ramachandran**					
Outliers	0.00%	0.02%	0.00%	0.04%	0.07%
Allowed	3.10%	2.51%	3.31%	2.90%	2.64%
Favored	96.90%	97.47%	96.69%	97.06%	97.29%

B

Dataset	RyR1, closed (EGTA only)	RyR1, closed	RyR1, open	RyR1, closed + simvastatin	RyR1, open + simvastatin
PDB ID	9NMR	9NMO	9NMN	9NMQ	9NMP
**Simvastatin:**					
**Sim-1**					
CC	N/A	N/A	N/A	0.79	0.76
RMSD:					
Bond lengths	N/A	N/A	N/A	0.004 Å	0.008 Å
Bond angles	N/A	N/A	N/A	2.341°	2.067°
**Sim-2**					
CC	N/A	N/A	N/A	0.70	0.66
RMSD:					
Bond lengths	N/A	N/A	N/A	0.006 Å	0.007 Å
Bond angles	N/A	N/A	N/A	2.317°	1.993°
**PC lipid:**					
**PCW-1**					
CC	0.65	0.75	0.73	0.63	0.63
RMSD:					
Bond lengths	0.039 Å	0.039 Å	0.039 Å	0.038 Å	0.039 Å
Bond angles	5.203°	5.150°	5.844°	5.996°	5.254°
**PCW-2**					
CC	0.54	0.69	0.64	0.59	0.57
RMSD:					
Bond lengths	0.039 Å	0.040 Å	0.039 Å	0.039 Å	0.039 Å
Bond angles	5.139°	5.817°	5.159°	5.714°	5.779°
**Zn^{2+}**					
CC	0.97	0.95	0.88	0.96	0.92
**Ca^{2+}**					
CC	N/A	0.82	0.81	0.83	0.85
**ATP (Core)**					
CC	N/A	0.76	0.73	0.78	0.79
RMSD:					
Bond lengths	N/A	0.003 Å	0.003 Å	0.004 Å	0.004 Å
Bond angles	N/A	1.906°	1.906°	1.953°	1.957°
**ATP (RY1&2)**					
CC	N/A	0.57	0.60	0.63	0.64
RMSD:					
Bond lengths	N/A	0.002 Å	0.002 Å	0.002 Å	0.002 Å
Bond angles	N/A	1.904°	1.908°	1.905°	1.883°
**Caffeine**					
CC	N/A	0.84	0.83	0.84	0.82
RMSD:					
Bond lengths	N/A	0.033 Å	0.033 Å	0.035 Å	0.034 Å
Bond angles	N/A	5.677°	5.781°	5.715°	5.788°

Supplementary Table 1. Cryo-EM data collection, processing parameters and model statistics for RyR1 structures with and without simvastatin. (**A**) RyR1 data sets. (**B**) Ligand correlation coefficients (CC) and RMSD values of bond lengths and bond angles (as determined in Phenix).

Mean ± SD	Placebo			Simvastatin			Simvastatin + S107		
Treatment week	0	4	6	0	4	6	0	4	6
pH	7.33 ± 0.08	7.35 ± 0.06	7.39 ± 0.04	7.36 ± 0.05	7.39 ± 0.05	7.31 ± 0.04	7.36 ± 0.04	7.39 ± 0.03	7.34 ± 0.03
PCO2 (mmHg)	33.6 ± 4.3	33.6 ± 5.0	29.0 ± 4.0	33.7 ± 4.3	27.7 ± 3.1	35.8 ± 3.2	32.4 ± 4.7	28.2 ± 3.2	32.0 ± 5.3
PO2 (mmHg)	59.3 ± 5.9	60.9 ± 11.1	67.7 ± 10.3	63.3 ± 11.0	74.5 ± 11.1	65.7 ± 8.6	63.6 ± 9.7	72.4 ± 10.8	70.8 ± 9.4
Beecf (mM)	-8.2 ± 3.6	-6.9 ± 2.0	-7.4 ± 1.4	-6.6 ± 2.2	-7.8 ± 2.1	-8.0 ± 2.9	-7.0 ± 2.3	-7.7 ± 2.1	-8.3 ± 2.5
HCO3 (mM)	17.6 ± 2.6	18.6 ± 1.8	17.5 ± 1.2	18.8 ± 1.9	16.9 ± 1.5	18.2 ± 2.2	18.4 ± 2.3	17.2 ± 1.8	17.3 ± 2.4
TCO2 (mM)	18.7 ± 2.5	19.6 ± 1.8	18.4 ± 1.3	19.9 ± 2.0	17.7 ± 1.6	19.4 ± 2.5	19.6 ± 2.6	18.0 ± 2.1	18.4 ± 2.7
SO2 (%)	87.9 ± 4.3	88.9 ± 4.3	92.4 ± 4.4	90.0 ± 4.2	94.2 ± 2.5	90.4 ± 3.1	90.0 ± 3.5	93.8 ± 3.0	92.8 ± 2.5
Na^+ (mM)	144.2 ± 1.3	147.4 ± 2.3	150.0 ± 2.1	145.4 ± 1.3	145.6 ± 1.3	153.3 ± 2.6	144.4 ± 1.3	144.9 ± 1.7	148.8 ± 3.0
K^+ (mM)	7.2 ± 1.0	7.1 ± 1.3	5.3 ± 0.6	6.8 ± 0.8	5.6 ± 1.1	6.2 ± 0.8	7.1 ± 1.0	6.4 ± 1.5	5.3 ± 0.5
iCa (mM)	1.09 ± 0.04	1.16 ± 0.04	1.17 ± 0.04	1.12 ± 0.02	1.15 ± 0.04	1.22 ± 0.03	1.11 ± 0.05	1.14 ± 0.05	1.19 ± 0.04
Glu (mg/dl)	195.7 ± 25.8	195.9 ± 13.1	204.1 ± 26.6	193.0 ± 31.1	212.3 ± 31.0	175.6 ± 28.8	203.2 ± 29.0	223.0 ± 28.2	194.9 ± 27.6
Hct (% PCU)	43.2 ± 6.9	45.8 ±1.8	46.5 ± 2.5	44.9 ± 3.5	44.3 ± 2.3	47.5 ± 1.9	45.7 ± 3.7	46.0 ± 1.8	48.5 ± 2.2
Hb (g/dl)	14.7 ± 2.3	15.6 ± 0.6	15.8 ± 0.9	15.3 ± 1.2	15.1 ± 0.8	16.2 ± 0.6	15.5 ± 1.3	15.7 ± 0.6	16.5 ± 0.7

Supplemental Table 2. Blood tests of WT mice treated with simvastatin. WT mice were treated with placebo, ~50 mg/kg/day simvastatin, or the latter together with ~50 mg/kg/day S107. Blood samples were taken before (week 0), in between (week 4), and after treatment (week 6). Gases and ions in the blood plasma were analyzed using *i-STAT* CG8+ cartridges. Data are expressed as mean ± SD. No significant changes were detected upon simvastatin treatment in the blood tests.
